# mRNA delivery of mosaic-8 pan-sarbecovirus RBD vaccines elicits distinct antibody epitope signatures

**DOI:** 10.1016/j.celrep.2026.117335

**Published:** 2026-04-30

**Authors:** Alexander A. Cohen, Jennifer R. Keeffe, Lusineh Manasyan, Indeever Madireddy, Ange-Célia I. Priso Fils, Kim-Marie A. Dam, Haley E. Stober, Rory A. Hills, Woohyun J. Moon, Paulo J.C. Lin, Mark R. Howarth, Magnus A.G. Hoffmann, Pamela J. Bjorkman

**Affiliations:** 1 Division of Biology and Biological Engineering, California Institute of Technology, Pasadena, CA 91125, USA; 2 Gladstone Institutes, San Francisco, CA 94158, USA; 3 Department of Pharmacology, University of Cambridge, Tennis Court Road, CB2 1PD Cambridge, UK; 4 Department of Biochemistry, University of Oxford, OX1 3QU Oxford, UK; 5 Acuitas Therapeutics, Vancouver, BC V6T 1Z3, Canada; 6 Department of Medicine, University, San Francisco, CA 94143, USA; 7 Present address: Department of Pathology, Stanford University, School of Medicine, Palo Alto, CA 94305, USA; 8 Lead contact

## Abstract

Protein-based mosaic-8 nanoparticles displaying eight SARS-like betacoronavirus (sarbecovirus) receptor-binding domains (RBDs) elicited broadly cross-reactive antibodies that could protect from zoonotic spillovers. Here, we extend the mosaic-8 concept to mRNA by encoding membrane-bound RBD quartets (four linked RBDs) as dual quartet RBD-mRNA and dual quartet RBD-EABR-mRNA, the latter leveraging ESCRT- and ALIX-binding region (EABR) technology for display on cell surfaces and secreted virus-like particles. Compared with protein-based mosaic-8, mRNA-encoded mosaic-8 induced equivalent or enhanced antibody breadth, neutralization potencies, and conserved epitope targeting, while eliciting enhanced T cell responses and more balanced IgG subclass profiles consistent with potentially superior Fc effector functions. Finally, systems serology-polyclonal epitope mapping (SySPEM) revealed distinct IgG-sub-class-specific epitope signatures across mRNA, EABR-mRNA, and protein vaccines, demonstrating that the mode of antigen display can shape epitope recognition. Successful conversion of a multivalent protein vaccine to mRNA platforms informs the design of broadly protective vaccines and advances mosaic-8 toward clinical development.

## In brief

Mosaic-RBD COVID-19 boosters could prevent SARS-like betacoronavirus spillovers from causing epidemics/pandemics, yet protein-based versions present manufacturing challenges and do not elicit strong CD8^+^ T cell responses. Cohen et al. convert a mosaic-8 RBD nanoparticle vaccine to an mRNA-LNP format, demonstrating equivalently broad antibody and superior T cell responses compared with protein-based counterparts.

## Graphical Abstract

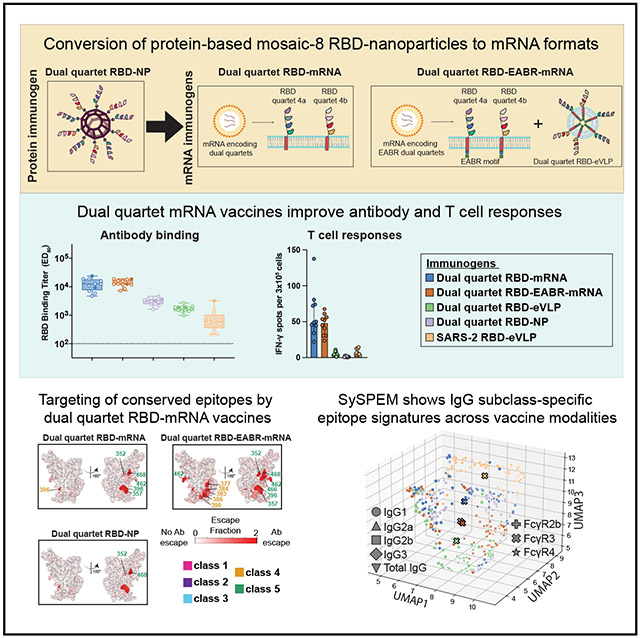

## INTRODUCTION

Three coronaviruses have spilled over from animal hosts to cause human epidemics or pandemics in the past 25 years: SARS-CoV (hereafter SARS-1), MERS-CoV, and SARS-CoV-2 (hereafter SARS-2).^1^ Two of these, SARS-1 and SARS-2, are members of the SARS-like betacoronavirus (sarbecovirus) subgenus. SARS-2 continues to infect humans, driving the emergence of new variants that necessitate regular updates of current COVID-19 vaccines.^2^ Since we cannot predict how SARS-2 will continue to mutate or which zoonotic sarbecovirus will next infect humans, we need a pan-sarbecovirus vaccine that does not require updating to provide broad protection against new SARS-2 variants and future sarbecovirus spillovers.^3^

**Figure 1.**
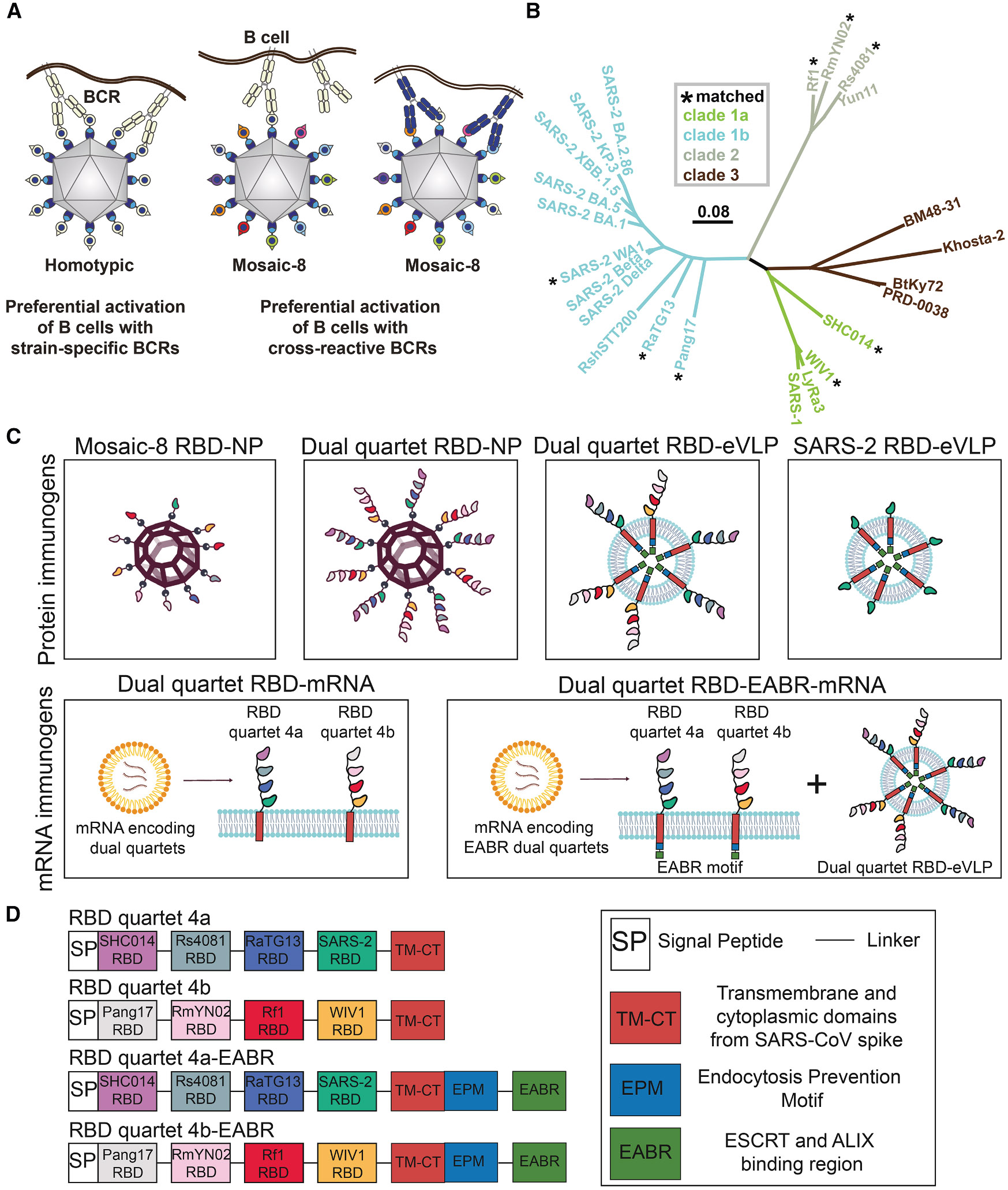
Protein- and mRNA-based pan-sarbecovirus immunogens (A) Avidity hypothesis. Left: membrane-bound strain-specific BCRs (pale yellow) using avidity to recognize a strain-specific epitope (pale yellow triangle) on antigens attached to a homotypic NP. Middle: strain-specific BCRs binding with only a single Fab (i.e., not using avidity) to a strain-specific epitope (triangle) on an antigen attached to a mosaic NP. Right: cross-reactive BCRs using avidity to recognize a common epitope (blue circle) presented on different antigens attached to a mosaic NP, but not to strain-specific epitopes (triangles). Only a fraction of the 60 attached RBDs are shown for clarity. (B) Sarbecovirus phylogenetic tree (made using a Jukes-Cantor generic distance model with Geneious Prime 2023.1.2) calculated from amino acid sequences of RBDs aligned using Clustal Omega.^14^ Viruses with RBDs included in RBD quartets and mosaic-8 RBD-NPs are indicated with an asterisk. Scale bars represent phylogenetic distance of 0.08 nucleotide substitutions per site. (C) Schematics of protein-based and mRNA-based pan-sarbecovirus immunogens (not to scale; showing only a fraction of antigens for clarity). (D) Schematics of mRNA constructs encoding RBD dual quartet immunogens used in this study.

Spike trimers of coronaviruses function in host cell entry after one or more receptor-binding domains (RBDs) adopt an “up” position to permit interactions with a host receptor.^4^ RBD targeting has been suggested for COVID-19 vaccine development, as RBDs are the main target of neutralizing antibodies (Abs).^5^ We previously described a vaccine approach that elicits broad Ab responses to diverse sarbecoviruses involving RBDs from multiple sarbecoviruses that are covalently linked using the SpyCatcher-SpyTag system^6^ to a SpyCatcher-mi3 protein nanoparticle (NP).^7-10^ In this approach, mosaic-8 RBD-NPs displayed eight different sarbecovirus spike RBDs attached randomly to the 60 positions of the mi3 NP (Figures 1A and 1B). We hypothesized that B cells bearing cross-reactive receptors (B cell receptors; BCRs) recognizing features in common between adjacent non-identical RBDs are preferentially activated compared to B cells with BCRs recognizing immunodominant strain-specific epitopes^8^ (Figure 1A). To investigate this, we used deep mutational scanning (DMS)^11^ to map RBD epitopes recognized by Abs from animals immunized with mosaic-8 RBD-NPs or homotypic RBD-NPs (only presenting SARS-2 RBD), revealing targeting of more conserved RBD regions by mosaic-8 RBD-NP-elicited Abs compared with targeting of more variable RBD regions by homotypic RBD-NP-elicited Abs.^8-10,12,13^ This finding rationalized results from challenge experiments in which mosaic-8 RBD-NPs protected from both a matched viral challenge (from a virus represented by an RBD on the NP) and a mismatched challenge (from a virus not represented by an RBD on the NP), whereas homotypic RBD-NPs protected only from a matched challenge.^8^ We also found broader Ab responses elicited by mosaic-8 RBD-NPs in animals that were pre-vaccinated with COVID-19 vaccines.^9^ These results suggest that a mosaic-8 RBD-based vaccine given as a COVID-19 booster could provide increased protection from SARS-2 variants and prevent future sarbecovirus spillover(s) from causing an epidemic or pandemic.

Manufacturing a mosaic-8 RBD-NP vaccine would require producing nine proteins: eight RBDs and an NP to which the RBDs are coupled. Making an mRNA-based version of a mosaic-8 vaccine would also pose challenges due to the requirement of synthesizing eight individual modified mRNAs. The RBD-encoding mRNAs could either be packaged into individual lipid NPs (LNPs) or co-formulated into a single LNP. In the first case, some cells would not take up all eight mRNA LNPs, therefore presenting fewer instances of different adjacent RBDs, potentially reducing avidity effects to activate B cells with maximally cross-reactive BCRs (Figure 1A). In the second case, co-formulation of eight mRNAs into a single LNP would present technical and regulatory challenges. A potential solution relevant to converting a mosaic-8 vaccine into an mRNA format involves making mi3 NPs presenting two RBD “quartets” (each containing four RBDs arranged in tandem), which elicit equally broad and potent Abs as the original mosaic-8 RBD-NP presenting monomeric RBDs^12^ (Figure 1C; dual quartet RBD-NP). Dual quartet RBD-NPs require the manufacture of only three components (two quartets and an NP) and, by synthesizing mRNAs encoding membrane-bound RBD quartets, could be more easily converted to an mRNA-LNP format than mosaic-8 RBD-NPs. In addition to simpler manufacturing, mRNA-LNP vaccines offer advantages over protein-based vaccines in that viral antigens undergo translation in the cytoplasm, thereby providing a source of viral peptides for presentation by MHC class I molecules to activate CD8^+^ cytotoxic T cells.^15^

Conversion of dual RBD quartet immunogens to an mRNA format (Figure 1C; dual quartet RBD-mRNA) is also compatible with use of the ESCRT- and ALIX-binding region (EABR) mRNA vaccine platform, which presents immunogens on cell surfaces and on circulating enveloped virus-like particles (eVLPs), thereby combining attributes of both mRNA and protein-NP vaccines^16^ (Figure 1C; dual quartet RBD-EABR-mRNA). eVLP formation is achieved by appending an EABR sequence to the cytoplasmic domain of the mRNA-encoded immunogen, which recruits cellular ESCRT proteins to induce eVLP budding from the plasma membrane. We previously showed that two immunizations with mRNA-LNPs encoding spike-EABR elicited robust CD8^+^ T cell responses and superior neutralizing Ab responses against original and variant SARS-2 compared to conventional spike-encoding mRNA-LNPs, improving neutralizing titers >10-fold against Omicron-based variants.^16^

Until this study, it was not known whether presentation of membrane-bound RBD quartets on eVLPs and/or the surfaces of cells would elicit cross-reactive Abs to the extent we observed using RBD quartets or single RBDs covalently linked to rigid protein NPs. Here, we show that the advantages of protein-based mosaic-8 RBD-NPs can be transferred to an mRNA-LNP format. We compared immune responses in mice immunized with dual quartet RBD-mRNA versions of mosaic-8 immunogens (dual quartet RBD-mRNA and dual quartet RBD-EABR-mRNA) with responses to two protein-based mosaic-8 NPs (dual quartet RBD-NPs^12^ and EABR-based dual quartet RBD-eVLPs) or to homotypic SARS-2 RBD-eVLPs. Immunization with mRNA immunogens elicited equivalent or improved binding breadths, neutralization potencies, T cell responses, and targeting of conserved RBD epitopes across sarbecoviruses compared to protein-based mosaic-8 immunogens, demonstrating successful conversion of the mosaic-8 RBD vaccine to mRNA formats that enable streamlined, scalable manufacturing of a pan-sarbecovirus vaccine.

## RESULTS

### mRNA-encoded RBD quartets are presented on cell surfaces and eVLPs

mRNA-LNPs direct cell surface expression of a variety of gene products, including viral antigens.^17^ We investigated whether protein-based soluble dual quartets could be modified to induce cell surface expression by creating constructs that encode an RBD quartet followed by a linker, transmembrane anchoring sequence, and cytoplasmic tail (Figure 1D; RBD quartets 4a and 4b). The eight RBDs in the 4a and 4b quartet sequences correspond to the RBDs used in previous studies of protein-based dual quartet RBD-NPs^12^ and to RBDs in a mosaic-8 RBD-NP constructed from soluble monomeric RBDs^7^ (Figure 1B). We used flow cytometry to verify presentation of membrane-bound RBD quartets on the surface of cells transfected with mRNA encoding RBD quartets 4a or 4b by staining with C118, a human monoclonal Ab (mAb) that recognizes a conserved RBD epitope present on all eight RBDs.^18,19^ Flow cytometry showed high and equivalent levels of RBD quartets 4a and 4b on cell surfaces when expressed separately (Figure S1A).

We next investigated the use of EABR technology, which modifies membrane proteins delivered by mRNA-LNPs to induce self-assembly and budding of eVLPs, resulting in immunogen presentation on cell surfaces and circulating eVLPs,^16^ for delivering quartet RBD sequences. eVLPs are induced by appending a short sequence to the cytoplasmic domain of a membrane protein, which recruits proteins from the host ESCRT pathway that drives budding in many enveloped viruses.^20^ Addition of an EABR sequence produces eVLPs for a variety of membrane proteins including SARS-2 spike,^16^ HIV-1 Env,^16^ influenza hemagglutinin,^21^ RSV fusion protein,^22^ Andes virus glycoprotein,^23^ CCR5,^16^ epidermal growth factor receptor,^24^ and MHC class I proteins.^25^ Compared to non-EABR RBD quartets, cells expressing EABR-modified RBD quartets showed lower cell surface expression (Figure S1A), which presumably results from a subset of cell surface proteins being incorporated into released eVLPs.

To evaluate whether the RBD quartet 4a-EABR and 4b-EABR constructs induced secretion of RBD quartet-displaying eVLPs, we examined eVLPs purified from supernatants of quartet 4a- or 4b-transfected cells. ELISAs were consistent with efficient eVLP formation for EABR mRNAs, showing high quartet 4a or 4b protein levels in purified eVLP samples for EABR, but not non-EABR, quartets (Figure S1B). We also used Abs specific for quartet 4a (anti-Rs4081 RBD)^26^ and 4b (anti-WIV1 RBD)^27^ to examine co-transfected cells by flow cytometry, demonstrating that RBD quartets 4a and 4b were co-expressed on individual cell surfaces (Figure S1C). To confirm that both quartets were incorporated into the same eVLP, we used a sandwich ELISA to probe eVLPs from an RBD quartet 4a-EABR and 4b-EABR co-transfection. eVLPs were purified, captured with anti-WIV1 RBD Ab (quartet 4b), and detected with anti-Rs4081 RBD Ab (quartet 4a), verifying the presence of both quartets on dual quartet eVLPs (Figure S1D). No binding was detected for samples prepared in the same way from cells co-transfected with non-EABR versions of the quartets or cells only transfected with a single quartet (Figure S1D). These data show that mRNA-encoded RBD quartets are co-expressed on transfected cells and that the two EABR-modified quartets are co-displayed on eVLPs.

### Dual quartet RBD mRNA immunogens elicit cross-reactive Abs

**Figure 2.**
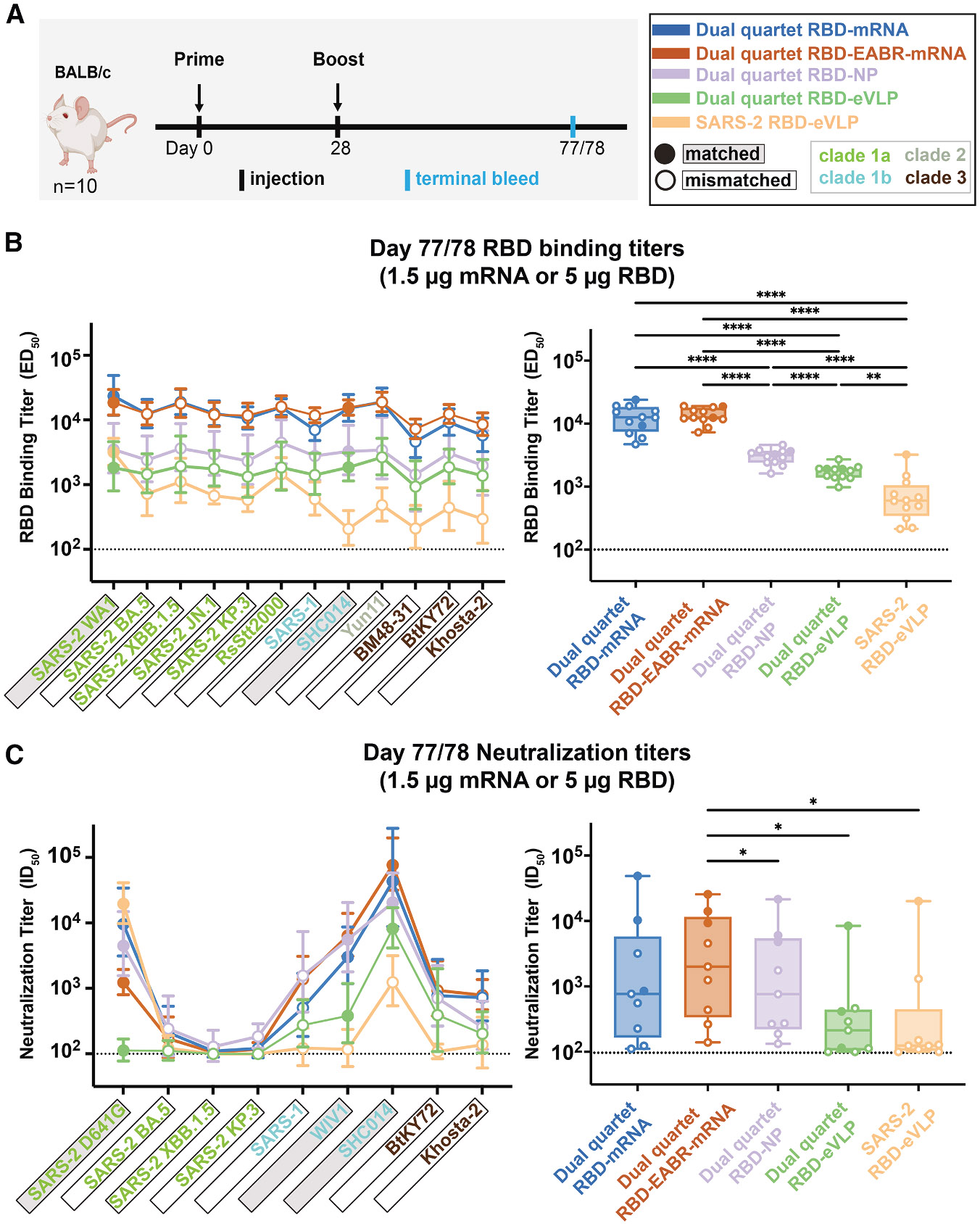
Dual quartet RBD mRNA immunogens elicit cross-reactive Abs (A) Immunization regimen. Left: mice were primed at day 0, boosted at day 28, and samples were collected from a terminal bleed at day 77 or 78. Right: colors used to identify immunization cohorts and symbols indicating a sarbecovirus antigen or pseudovirus that is matched (filled in data points; gray shading around name) or mismatched (unfilled data points; black outline around name). Sarbecovirus strain names are colored in (B) and (C) according to clade. (B and C) Results for RBD-binding ELISAs (B) and pseudovirus neutralization assays (C) for serum samples from day 77 or 78. Dashed horizontal lines indicate detection limits for assays. Left: geomeans of RBD-binding ELISA half-maximal effective dilution (ED_50_) values (B) or neutralization half-maximal inhibitory dilution (ID50) values (C) across a panel of viral antigens or pseudoviruses for sera from animals in each cohort (geomeans are plotted as symbols with geometric standard deviations indicated by error bars). Mean ED_50_ or ID_50_ values (*n* = 10 sera) across antigens are connected by colored lines corresponding to the immunization cohort. Right: box and whisker plots of geomean responses (ELISA ED50s in B, *n* = 11 antigens; neutralization ID50s in C, *n* = 9 pseudoviruses) with individual data points representing geomean responses to a single antigen or pseudovirus as shown in the left. Boxes display the range between the upper and lower quartiles, with a line denoting the median value. Whiskers extend to minimum and maximum values, excluding any outliers. Geomean titers were compared pairwise between immunization cohorts by Tukey’s multiple comparison test with the Geisser-Greenhouse correction (as calculated by GraphPad Prism). Significant differences between cohorts are indicated by asterisks: *p < 0.05, **p < 0.01, ***p < 0.001, and ****p < 0.0001. ELISA and neutralization results for terminal bleed serum samples (see also Figure S2).

We next compared Ab responses in mice immunized with mRNA-encoded dual quartets to immunizations with protein-based dual quartet RBD-NPs or with purified dual quartet RBD-eVLPs. mRNAs encoding RBD quartets 4a and 4b were co-formulated into LNPs to generate dual quartet immunogens (dual quartet RBD-mRNA and dual quartet RBD-EABR-mRNA), which were each injected intramuscularly at RNA doses of 1.5 or 0.5 μg (Figures 2A and S2A). We also included cohorts of mice that were immunized with 5 μg of dual quartet RBD-NPs (calculated based on RBD content) in the presence of adjuvant (Figure 2A) and cohorts immunized with adjuvanted purified RBD-eVLPs (dual quartet RBD-eVLP or SARS-2 RBD-eVLP; 5 μg RBD content per dose), the latter representing a homotypic immunogen control presenting only one type of RBD (Figure 1C).

Since binding and neutralizing Ab responses correlate with protection in humans and animals vaccinated with COVID-19 mRNA vaccines,^28-30^ we analyzed immunized mouse sera by ELISA and pseudovirus assays^31^ to evaluate Ab binding responses and neutralization, respectively (Figures 2B, 2C, and S2B). When comparing mRNA-based to protein-based immunogens, we used a 1.5 μg mRNA dose and a 5 μg (RBD content) dose for the protein-based immunogens (Figures 2B and 2C), representing standard doses in mice.^7,16,32^ We also compared the mRNA and mRNA-EABR immunogens using a 0.5 μg mRNA dose to investigate potential dose-sparing effects (Figure S2B). To evaluate sarbecovirus strain-specific differences in Ab binding and neutralization properties, we displayed data showing responses to individual sarbecovirus antigens (Figures 2B and 2C, left) as well as overall geometric mean (geomean) responses elicited by different cohorts across the evaluated antigens (Figures 2B and 2C, right).

Highest mean binding titers for sera across a panel of matched and mismatched sarbecovirus RBDs were found for dual quartet RBD-mRNA and dual quartet RBD-EABR-mRNA cohorts (Figure 2B, left). Thus, genetically encoded RBD quartets delivered by a 1.5 μg dose of mRNA-LNPs were significantly more effective at eliciting cross-reactive RBD-binding Abs across a panel of sarbecovirus RBDs than 5 μg of the dual quartet RBD-NP protein-based immunogen (Figure 2B, right; *p* < 0.0001 for comparisons of dual quartet RBD-mRNA and dual quartet RBD-EABR-mRNA). In addition, both dual quartet RBD-mRNA and dual quartet RBD-EABR-mRNA exhibited significantly higher geomean binding titers than the protein-based dual quartet RBD-eVLPs and homotypic SARS-2 RBD-eVLPs (Figure 2B, right; *p* < 0.0001 for all comparisons of mRNA-LNP and protein-based immunogens). SARS-2 RBD-eVLPs exhibited significantly lower binding titers across the RBD panel than the dual quartet RBD-NPs and purified dual quartet RBD-eVLPs (Figure 2B, right; *p* < 0.0001 for all comparisons except *p* = 0.0057 for dual quartet RBD-eVLP), as expected based on results for homotypic RBD-NPs.^7,8^

We next compared neutralizing titers across a panel of matched and mismatched sarbecovirus strains (Figure 2C, left). When comparing by strain across the five immunization cohorts, dual quartet RBD-mRNA, dual quartet RBD-EABR-mRNA, and dual quartet RBD-NP exhibited the highest titers across the pseudovirus panel except for neutralization of SARS-2D614G, where homotypic SARS-2 RBD-eVLPs elicited slightly higher titers (Figure 2C, left). Geomean neutralization titers for the five cohorts (Figure 2C, right) were more similar to each other than the mean binding titers (Figure 2B, right), but dual quartet RBD-EABR-mRNA induced significantly higher mean neutralizing titers than dual quartet RBD-NP (*p* = 0.0152), dual quartet RBD-eVLP (*p* = 0.0152), and SARS-2 RBD-eVLP (*p* = 0.0470).

We also measured binding and neutralization titers elicited by 0.5 μg doses of dual quartet RBD-mRNA and dual quartet RBD-EABR-mRNA (Figures S2A and S2B). Binding titers against the RBDs tested were not statistically different for the two mRNA immunogens, except for binding to the BM48-31 RBD, in which case dual quartet RBD-EABR-mRNA induced significantly higher binding than dual quartet RBD-mRNA (Figure S2B, left; *p* = 0.0089). Neutralization titers were significantly different for three viruses: SARS-2D614G (titers higher for dual quartet RBD-mRNA; *p* = 0.00013) and for SARS-1 and WIV1 (higher for dual quartet RBD-EABR-mRNA; *p* = 0.00024 and *p* < 0.0001, respectively) (Figure S2B, right). These results are consistent with more potent neutralization of non-SARS-2 viruses induced by dual quartet RBD-EABR-mRNA compared with dual quartet RBD-mRNA at the 1.5 μg dose (Figure 2C).

### mRNA-encoded dual quartets elicit robust T cell responses

T cell responses to the various immunogens were evaluated on day 77/78 in immunized mouse splenocytes by enzyme-linked immunospot (ELISpot) assays, a sandwich immunoassay in which cytokines secreted by lymphocytes are captured by membrane-bound Abs and then visualized as spots.^33^ Cytokines (IFN-γ produced by CD4^+^ T helper 1 [T_H_1] cells and cytotoxic CD8^+^ T cells; IL-4 produced by CD4^+^ T helper 2 [T_H_2] and T follicular helper cells^34^) were measured after stimulation with a pool of SARS-2 spike peptides.

**Figure 3.**
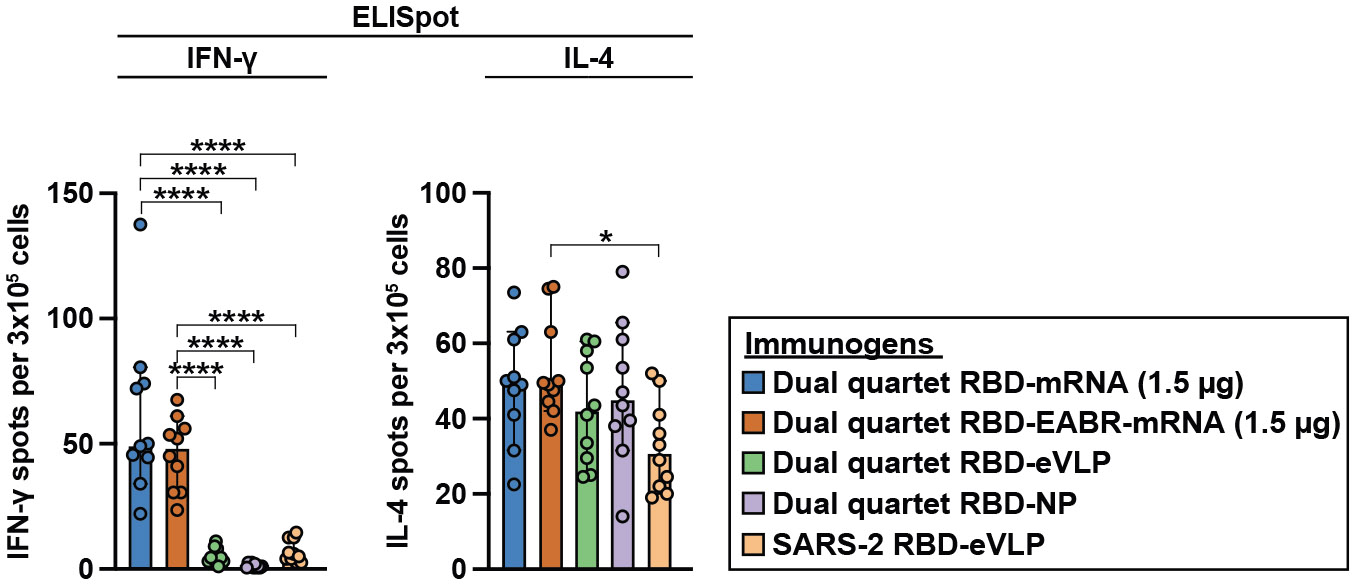
mRNA-encoded dual quartets elicit robust T cell responses ELISpot assay data for SARS-2 RBD-specific IFN-γ (left) and IL-4 (right) responses of splenocytes from BALB/c mice that were immunized with the indicated immunogens (immunization regimen in Figure 2A). Results are shown as spots per 3 × 10^5^ cells for individual mice (colored circles, *n* = 10 mice) presented as the median (bars) and standard deviation (error bars). Cohorts were compared by Tukey’s multiple comparison test calculated by GraphPad Prism. Significant differences between cohorts linked by horizontal lines are indicated by asterisks: *p < 0.05 and ****p < 0.0001.

Dual quartet RBD-mRNA and dual quartet RBD-EABR-mRNA cohorts exhibited robust IFN-γ responses, consistent with activation of RBD-specific cytotoxic CD8^+^ T cells, whereas low/undetectable IFN-γ responses were found for the protein-based dual quartet RBD-NP, dual quartet RBD-eVLP, and SARS-2 RBD-eVLP cohorts, consistent with previous findings for mice immunized with purified spike-EABR eVLPs^16^ and the fact that mRNA-LNPs, but not protein immunogens, promote intracellular expression of antigens that serve as sources for peptides loaded onto MHC class I proteins.^35^ We found strong IL-4 responses for both the mRNA and protein-based immunogen cohorts (Figure 3).

Thus, mRNA-based mosaic-8 dual RBD quartet immunizations induce superior (IFN-γ) or equivalent (IL-4) release of cytokines compared with protein-based mosaic-8 immunogens.

### mRNA-encoded and protein-based dual quartets elicit Abs targeting similar epitopes

**Figure 4.**
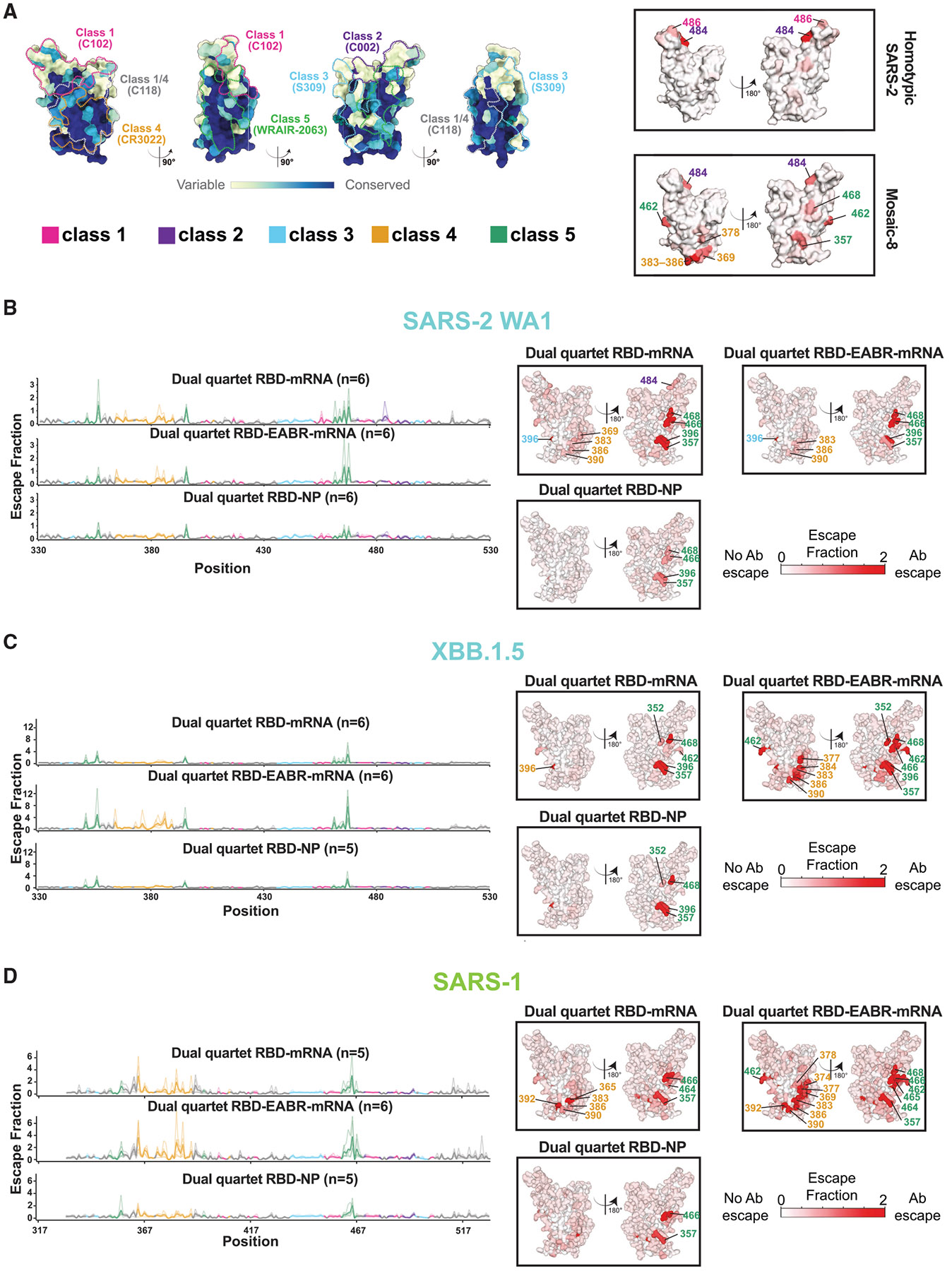
Dual quartet RBD-mRNA immunogens elicit Ab responses against conserved class 4 and class 5 RBD epitopes A) Left: sequence conservation of 516 sarbecovirus RBDs calculated using ConSurf^45^ shown on a surface representation of SARS-2 RBD (PDB: 7BZ5). Class 1, 2, 3, 4, 1/4, and 5 anti-RBD Ab epitopes^19,42-44^ are outlined in dots in different colors using information from representative structures of Abs bound to SARS-2 spike or RBD (C102: PDB: 7K8M; C002: PDB: 7K8T; S309: PDB: 7JX3; CR3022: PDB: 7LOP; C118: PDB: 7RKV; WRAIR-2063: PDB: 8EOO). Right: published DMS results^8^ mapped onto the surface of the SARS-2 WA1 RBD (PDB: 6M0J) for mosaic-8 RBD-NP and homotypic SARS-2 RBD-NP immunizations. (B–D) Left: line plots for DMS results using a SARS-2 WA1 RBD library (B), SARS-2 XBB.1.5 RBD library (C), and SARS-1 library (D) from the indicated number of mice immunized with the immunogens listed above each line plot. *x* axis: RBD residue number. *y* axis: sum of the Ab escape of all mutations at a site (larger numbers = more Ab escape). Each line represents one antiserum with thick lines showing the average across the *n* = 5 or 6 sera in each group. Lines are colored according to RBD epitopes in (A). Right: average site-total Ab escape calculated for results from *n* = 5 or 6 serum samples for the indicated RBD yeast display libraries. Mice were immunized with the immunogens listed, and results were mapped to the highlighted residues on the surface of the SARS-2 WA1 RBD (PDB: 6M0J). Gray indicates no escape; a gradient of red represents increasing degree of escape. Residue numbers show sites with the most escape with font colors representing different RBD epitopes (defined in A; class 1/4 residues are colored with fonts corresponding to class 1 or class 4 residues). The same data are shown in Figures S3-S6 (line and logo plots for SARS-2 WA1, SARS-2 XBB.1.5, and SARS-1 RBD libraries, and tabulated responses, respectively).

To map RBD epitopes targeted by Abs elicited by different vaccine modalities, we used DMS to evaluate the effects of all possible RBD amino acid changes on binding to polyclonal serum Abs.^11,36-41^ We classified RBD residues by epitopes defined by structural properties and conservation/variability across sarbecoviruses: class 1 and class 2 RBD epitopes exhibit higher, and class 4, class 1/4, and portions of class 3 and class 5 epitopes exhibit lower sequence variability across sarbecoviruses and SARS-2 variants^19,42-44^ (Figure 4A). We analyzed serum using yeast display libraries encoding RBDs derived from SARS-2 WA1 (matched), SARS-2 XBB.1.5 (mismatched), and SARS-1 (mismatched) spikes.

DMS showed that the epitopes targeted in the three RBD libraries by the mRNA-encoded dual quartet RBD immunogens were similar to each other and to epitopes targeted by the protein-based dual quartet RBD-NP immunogen (Figures 4A-4D and S3-S6). The dual quartet immunogens, like mosaic-8 RBD-NPs,^8,9^ did not primarily elicit Abs against the immunodominant and variable class 1 and class 2 RBD epitopes, as observed for Abs elicited by homotypic SARS-2 RBD NPs^8,9^ and spike-based mRNA vaccines.^46^ Instead, elicited Abs primarily targeted class 4 and class 5 RBD epitopes, except for the Abs raised by immunization with dual quartet RBD-NPs, where class 5 was the predominant targeted epitope.

Although dual quartet RBD immunizations resulted in a mixed class 4/class 5 RBD response when evaluated against the SARS-2 WA1, SARS-2 XBB.1.5, and SARS-1 RBD libraries (Figures 4B-4D and S3-S6), there were notable differences. For example, both RNA-based immunogens elicited similar class 4 and class 5 RBD responses, but dual quartet RBD-mRNA sera showed more of a class 1/class 2 RBD response than dual quartet RBD-EABR-mRNA sera against the SARS-2 WA1 RBD library (Figures 4B, S3, and S6), perhaps explaining why dual quartet RBD-mRNA elicited higher neutralization titers against the matched SARS-2D614G pseudovirus (Figures 2C and S2B). The dual quartet RBD-EABR-mRNA immunogen elicited a strong class 4 and class 5 response recognizing SARS-2 XBB.1.5 RBD, while the dual quartet RBD-mRNA and the RBD-NP immunogens elicited primarily a class 5 response against this SARS-2 variant (Figures 4C, S4, and S6). Against the SARS-1 RBD library, each of the dual quartet immunogens induced strong class 4 and class 5 responses. In addition, weak escape by Abs against the variable class 1 and class 2 RBD epitopes was seen for dual quartet RBD-mRNA, dual quartet RBD-EABR-mRNA, and dual quartet RBD-NP (Figures S5 and S6). Finally, one mouse in the dual quartet RBD-EABR-mRNA group and two in the dual quartet RBD-NP group elicited “polyclass” responses (defined as weak escape profiles that lacked clear features, which we interpret as resulting from sera containing multiple classes of anti-RBD Abs,^9^ consistent with the ability of these immunogens to induce Abs against multiple RBD epitopes.

We conclude that converting dual quartet RBD immunogens into an mRNA format retains the ability of mosaic-8 vaccines to elicit Abs mainly against more conserved RBD regions, as previously observed in DMS experiments comparing protein NPs presenting dual RBD quartets or eight individual RBDs.^12^

### mRNA-encoded dual quartet RBD immunogens elicit balanced IgG2a/IgG1 responses

IgG subclasses induce distinct Fc effector functions, including opsonization, phagocytosis, and Ab-dependent cell-mediated cytotoxicity, through differential binding of Fcs to Fc gamma receptors (FcγRs)^47^; e.g., mouse IgG1, IgG2a, and IgG2b bind the activating receptor FcγR3 and the inhibitory receptor FcγR2b; IgG2a and IgG2b bind the activating receptor FcγR4; and mouse IgG3 does not bind detectably to these FcγRs.^48^ FcγR3 is more widely expressed than FcγR4, with FcγR4 mainly expressed on monocytes/macrophages and neutrophils and FcγR3 found on those cells as well as dendritic cells, natural killer cells, basophils, mast cells, and eosinophils.^48^ In immunized mice, IgG2a is associated with T_H_1-type immune responses, effective activation of complement, and protective interactions with FcγRs, which are critical functions for clearing viruses.^49^ IgG1, although usually efficient in neutralizing viruses, is associated with T_H_2-type responses thought to be less effective than T_H_1 responses in combatting viruses.^50^

**Figure 5.**
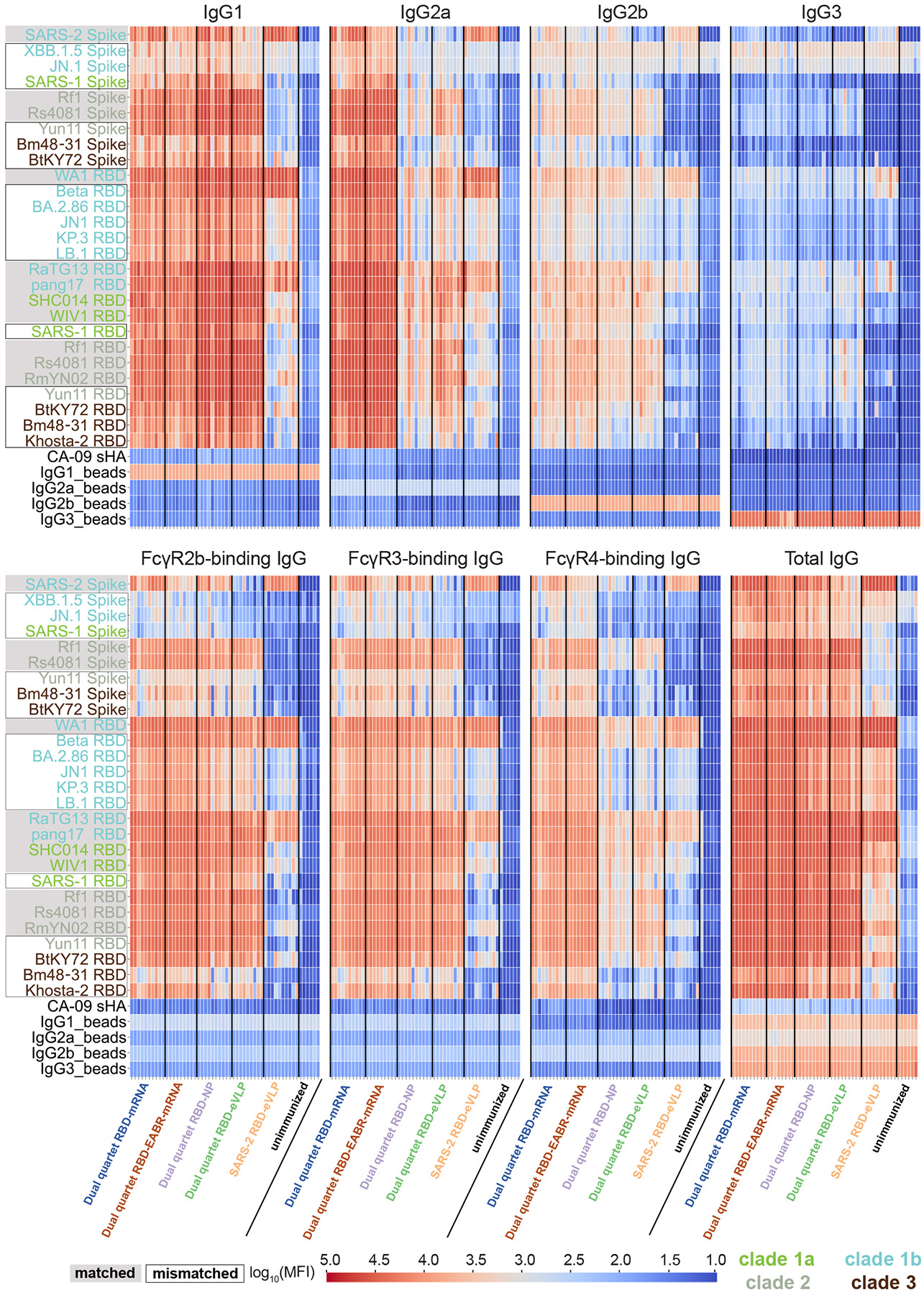
mRNA-encoded dual quartet RBD immunogens elicit balanced IgG subclass and potent FcγR-binding responses MFI, median fluorescent intensity. Binding of IgG1, IgG2a, IgG2b, IgG3, FcγR2b-binding IgGs, FcγR3-binding IgGs, FcγR4-binding IgGs, and total IgG to the indicated spikes, RBDs, or non-sarbecovirus control proteins. Antigen names (*y* axes) are colored according to spike or RBD clades. Matched antigens are indicated with gray shading around the name, and mismatched antigens are indicated with a black outline around the name. Each column represents binding data from an individual mouse, and immunization cohorts are separated by a vertical black line. A soluble influenza hemagglutinin trimer (CA-09 sHA) was used as a control to evaluate non-specific binding. See also Figure S7.

We used systems serology^51^ to evaluate IgG distributions elicited by mRNA versus protein immunogens by comparing binding of IgG1, IgG2a, IgG2b, IgG3, FcγR2b-binding IgGs, FcγR3-binding IgGs, FcγR4-binding IgGs, and total IgG (IgGs isolated using a pan-IgG Ab) elicited by the five immunogens to a panel of spike trimers and RBDs derived from SARS-2 variants and other sarbecoviruses. Results show elicited responses from individual mice to each antigen (Figure 5) and geomean responses across antigens for each immunogen (Figure S7).

Both dual quartet RBD-mRNA immunogens elicited high levels of IgG1 and IgG2a against sarbecovirus spikes and RBDs, with somewhat higher levels of IgG2a than IgG1 (Figures 5 and S7), demonstrating a desirable mixture of IgGs for an anti-viral vaccine inducing T_H_1 and T_H_2 responses. In addition, the mRNA-based immunogens elicited significantly higher IgG2a and FcγR4-binding IgG responses than the protein-based immunogens (*p* < 0.0001) (Figure S7). Dual quartet RBD-NPs and dual quartet RBD-eVLPs elicited higher levels of IgG1 than IgG2a, consistent with their administration with AddaVax adjuvant, which enhances T_H_2-biased immune responses.^52^ Dual quartet RBD-eVLPs also elicited significantly higher IgG2a than dual quartet RBD-NPs (*p* < 0.0001). Interestingly, dual quartet RBD-EABR-mRNA elicited significantly higher IgG2b (*p* < 0.0001), as well as FcγR3-binding IgGs (*p* = 0.0013) and FcγR4-binding IgGs (*p* = 0.0021), than dual quartet RBD-mRNA. Consistent with demonstrations of more limited sarbecovirus recognition properties of Abs raised in response to homotypic RBD-NPs,^7-9,26^ SARS-2 RBD-eVLP elicited IgGs mainly against SARS-2 and closely related RBDs (Figure 5).

Thus, mRNA versions of dual RBD quartets elicit balanced IgG responses that broadly recognize sarbecovirus antigens, arguing for their likely efficacy to protect against diverse sarbecoviruses.

### Systems serology-polyclonal epitope mapping

**Figure 6.**
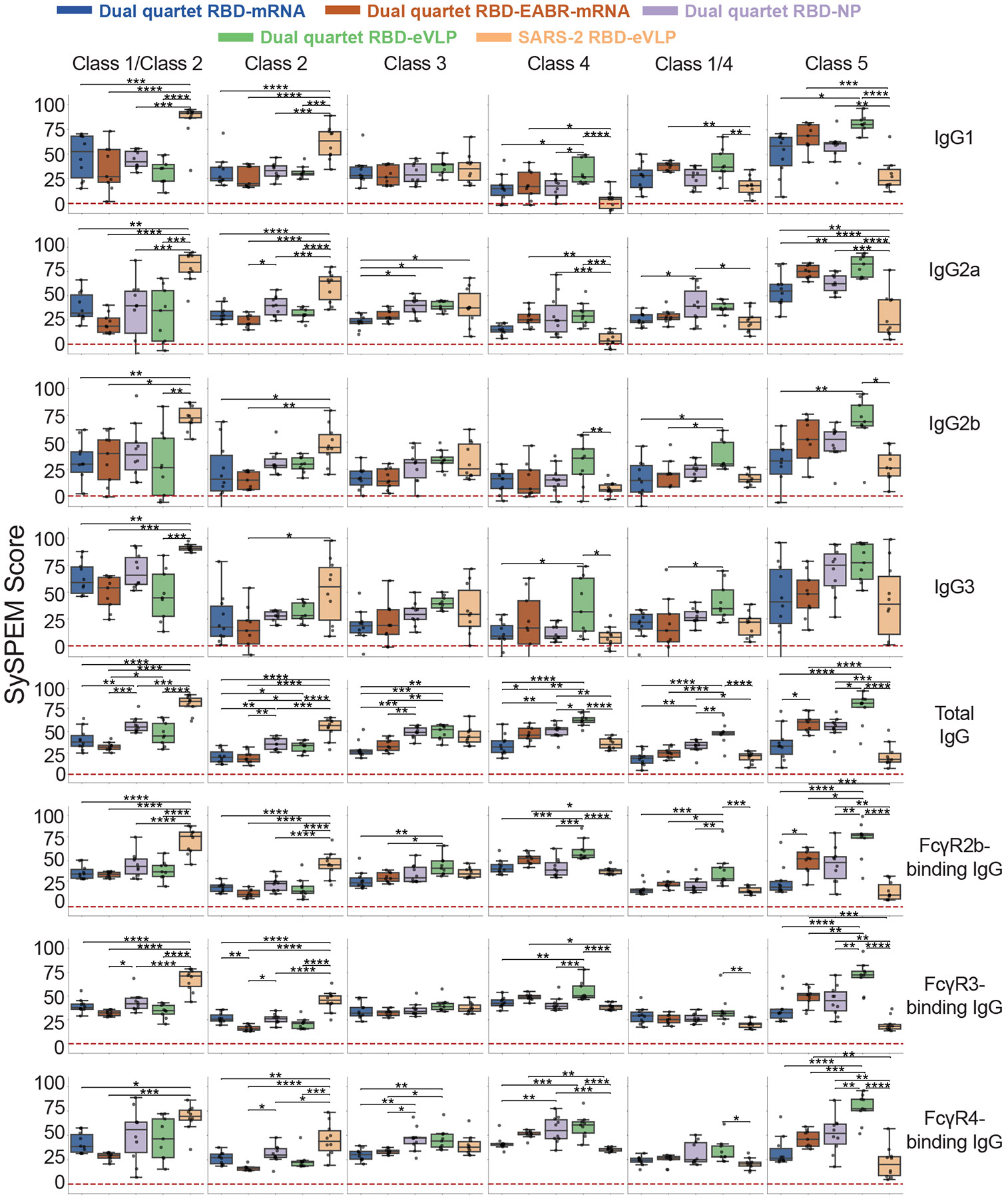
SySPEM score comparisons show differences in epitope recognition across immunogen cohorts SySPEM scores from individual mice were determined for RBD epitopes (columns) recognized by different IgG classes (rows, IgG subclass plus different FcγR-binding IgGs) with statistical comparisons between immunogen cohorts (colors). A SySPEM value of 0 indicates that none of the IgGs in that sample were affected by the glycan addition, and therefore, the sample did not contain IgGs that recognize that epitope, and a SySPEM value of 100 indicates that all IgGs in that sample recognized that epitope (Figure S8). A SySPEM value between 0 and 100 indicates the proportion of IgGs in a sample that recognized the epitope that was blocked by glycan addition in the RBD KO mutant. Box and whisker plots of SySPEM scores with individual data points representing one mouse are shown. Boxes display the range between the upper and lower quartiles, with a line denoting the median value. Whiskers extend to minimum and maximum values, excluding any outliers. Significant differences between cohorts were calculated using Tukey’s HSD post hoc test and linked by horizontal lines indicated by asterisks: *p < 0.05, **p < 0.01, ***p < 0.001, and ****p < 0.0001. See also Figures 7 and S10.

**Figure 7.**
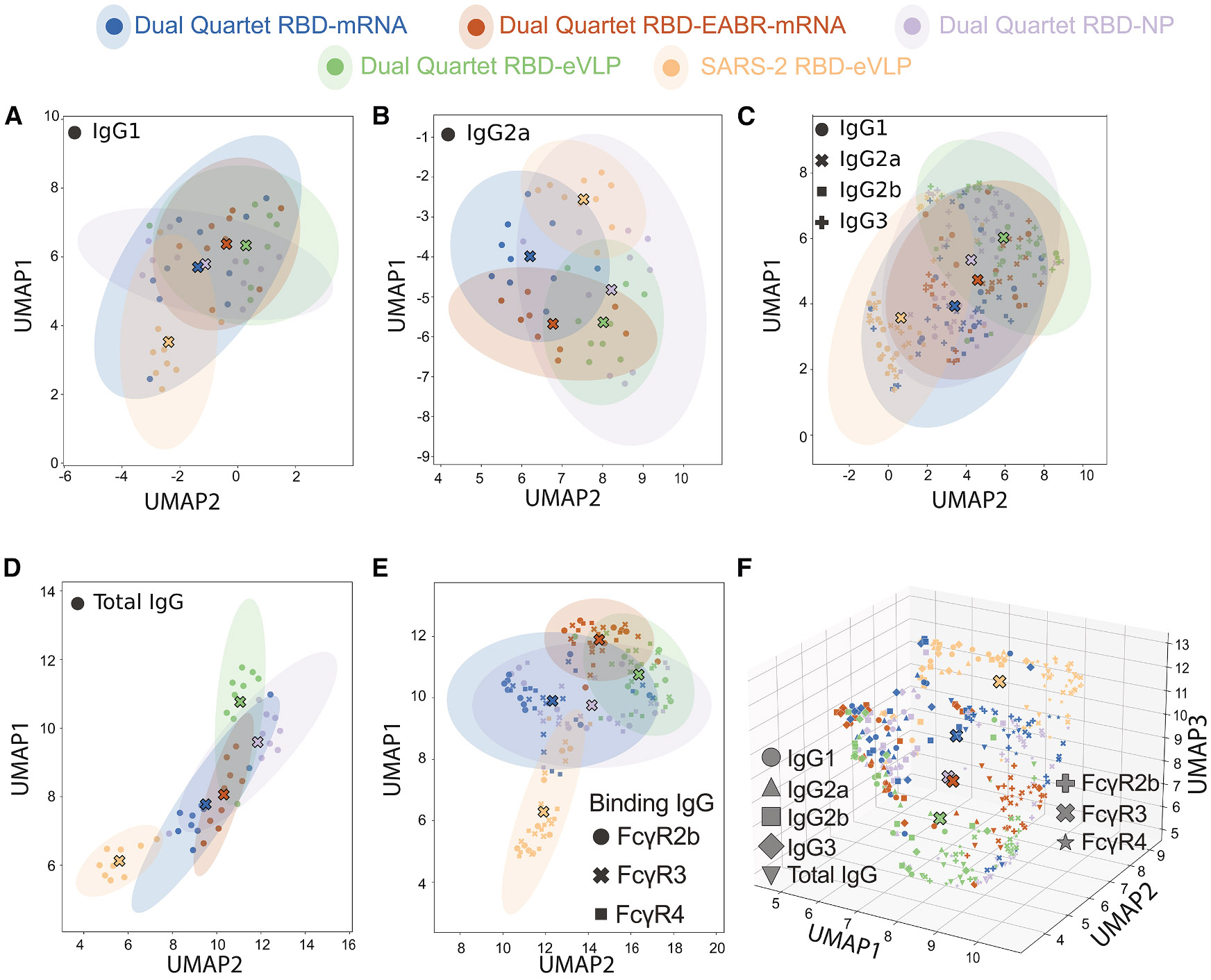
SySPEM UMAP analyses Uniform manifold approximation and projection (UMAP)^54^ was used to project multi-dimensional SySPEM scores into two (A–E) or three (F) dimensions for (A) IgG1, (B) IgG2a, (C) IgG1, IgG2a, IgG2b, and IgG3, (D) total IgG, (E) FcγR-binding IgGs, and (F) total IgG, FcγR-binding IgGs, and IgG subclasses. Samples with similar Ab-epitope recognition profiles cluster together, with colored ellipses indicating the variance within each immunogen group and centroid markers (large Xs) showing the group mean. (F) Three-dimensional UMAP projection for all IgG subclasses and FcγR-binding IgGs with centroid markers (large Xs). See also Figures Figure 6, S8, and S10

To further explore potential differences in RBD recognition properties by different IgG subclasses within polyclonal Ab samples, we combined systems serology with epitope mapping in a methodology we call systems serology-polyclonal epitope mapping (SySPEM) (Figure S8), by analogy to electron microscopy-based polyclonal epitope mapping (EMPEM).^53^ For epitope mapping by SySPEM, we created epitope knockout (KO) mutants in the SARS-2 WA1 RBD by introducing one or more potential N-linked glycosylation sites (PNGSs) to direct addition of N-glycan(s) in RBD epitopes that are recognized by either class 1 and class 2, class 2 only, class 3, class 1/4, class 4, or class 5 anti-RBD mAbs (Figure S9A). Addition of N-glycan(s) at introduced PNGS(s) was verified by SDS-PAGE of purified RBD KO proteins, and correct folding and epitope blocking were verified using a panel of control mAbs (Figures S9B and S9C). We measured binding of total polyclonal IgG and IgG subsets to unmodified (wild type; WT) WA1 RBD and to each RBD KO mutant, defining a SySPEM score for an IgG sample/epitope pair as (1.0 – [RBD KO binding/RBD WT binding] × 100); thus, higher SySPEM scores indicate increased binding by a particular IgG class to that epitope (Figure S8). We present SySPEM results in three formats to visualize epitope recognition across immunogens and Ab subclasses: (1) median SySPEM scores from individual mice divided into distinct RBD epitopes recognized by different IgG classes with statistical comparisons between immunogen cohorts (Figure 6). This comparison highlights differences in epitope recognition across immunogen cohorts. (2) Median SySPEM scores for each IgG class within a given immunogen cohort with statistical comparisons between targeted epitopes (Figure S10). This shows the epitope distribution for a given immunogen/IgG class pair, allowing epitope profile comparisons between different immunogens, analogous to a DMS profile (Figure 4). (3) Uniform manifold approximation and projection (UMAP)^54^ format to reduce the dataset dimensions (Figure 7). These figures provide hierarchical views of the SySPEM data: statistical comparisons across immunogens (Figure 6), detailed within-cohort analyses (Figure S10), and global similarity mapping of Ab recognition patterns (Figure 7).

SySPEM scores validated the mapping approach using RBD KO mutants by showing significantly higher scores for total IgGs elicited by homotypic SARS-2 RBD-eVLPs than by other immunogens against variable epitopes (class 1/class 2, class 2) and little to no targeting of more conserved epitopes (class 4, class 1/4, and class 5) (Figure 6), as previously observed by DMS for homotypic SARS-2 RBD-NPs.^8^

For IgG1, the mRNA- and protein-based dual quartet immuno-gens showed the highest class 5 SySPEM scores, with only dual quartet RBD-mRNA exhibiting a score that was not significantly higher than that of homotypic SARS-2 RBD-eVLPs (Figure 6). In addition, dual quartet RBD-eVLPs and dual quartet RBD-EABR-mRNA exhibited class 4 and class 1/4 IgG1 SySPEM scores that were significantly higher than respective scores for SARS-2 RBD-eVLPs. Similarly, for IgG2a, the dual quartet immunogens all elicited significantly higher class 5 responses than homotypic SARS-2 RBD-eVLP, with both dual quartet RBD-EABR-mRNA and dual quartet RBD-eVLP featuring the highest class 5 SySPEM scores. Dual quartet RBD-EABR-mRNA, dual quartet RBD-NP, and dual quartet RBD-eVLP also elicited higher class 4 SySPEM scores than SARS-2 RBD-eVLP. Although not significant, dual quartet RBD-mRNA elicited a higher class 1/class 2 SySPEM score than dual quartet RBD-EABR-mRNA, and the opposite trend was observed for class 5. These differences might explain the more potent SARS-2D614G neutralization elicited by dual quartet RBD-mRNA compared to dual quartet RBD-EABR-mRNA (Figure 2C), since class 1 and class 2 anti-RBD Abs are potent against SARS-2 WA1.^42^ However, the combination of higher class 5 and lower class 1/class 2 responses elicited by the dual quartet RBD-EABR-mRNA immunogen represents a more favorable epitope targeting profile for broad pan-sarbecovirus vaccine efforts. For IgG2b and IgG3, SySPEM scores were more similar across the different RBD epitope categories, although dual quartet RBD-eVLP showed significantly higher class 4 and class 5 SySPEM scores than SARS-2 RBD-eVLP.

For total IgG, dual quartet RBD-EABR-mRNA and dual quartet RBD-NP showed higher class 5 SySPEM scores than SARS-2 RBD-eVLP, with dual quartet RBD-eVLP showing the highest scores, and dual quartet RBD-mRNA showing class 5 scores that were not significantly higher than those exhibited by SARS-2 RBD-eVLP (Figure 6). Dual quartet RBD-EABR-mRNA exhibited significantly higher class 4 and class 5 SySPEM scores for total IgG than dual quartet RBD-mRNA, likely driven by the stronger class 4 and class 5 targeting by IgG2a for this comparison (Figure 6). Class 4 responses were higher for dual quartet RBD-EABR-mRNA, dual quartet RBD-NP, and dual quartet RBD-eVLP all exhibiting significantly higher class 4 SySPEM scores than SARS-2 RBD-eVLP, with dual quartet RBD-eVLP showing the highest class 4, 1/4, and 5 scores.

FcγR2b- and FcγR3-binding IgG SySPEM scores were similar across the RBD epitopes, with dual quartet RBD-EABR-mRNA, dual quartet RBD-NP, and dual quartet RBD-eVLP all exhibiting significantly higher class 5 SySPEM scores than SARS-2 RBD-eVLP (Figure 6). Dual quartet RBD-eVLP and, to a lesser extent, dual quartet RBD-EABR-mRNA exhibited higher class 4 SySPEM scores than SARS-2 RBD-eVLPs. For FcγR4-binding IgGs, dual quartet RBD-EABR-mRNA, dual quartet RBD-NP, and dual quartet RBD-eVLP showed significantly higher class 4 and class 5 SySPEM scores than SARS-2 RBD-eVLP.

To identify all epitopes recognized by sera elicited by a given immunogen, we displayed the SySPEM results in a format in which SySPEM scores for recognition of individual RBD epitopes by different IgG classes are shown (Figure S10). These results highlight increased targeting of class 5 RBD epitopes by IgG1, IgG2a, IgG2b, and IgG3 subclasses elicited by the four dual quartet immunogens but not by homotypic SARS-2 RBD-eVLPs. Displaying the data in this format showed that in some cases for a given immunogen, different IgG subclasses can exhibit distinct epitope profiles, e.g., IgG1 and IgG2a from dual quartet EABR-mRNA sera, where IgG1 showed higher class 1 and class 2 targeting than IgG2a, for which only class 5 targeting was high.

To visualize global patterns of epitope recognition between the different immunogens, we made UMAPs^54^ to project epitope profiles into two- or three-dimensional plots (Figure 7). For 2D UMAPs (Figures 7A-7E), clusters of points indicate samples that behaved similarly across all epitope KOs, with ellipses indicating the spread of responses from each immunogen and centroid markers (indicated by an X) indicating the average epitope mapping profile for each immunogen. When comparing IgG1 (Figure 7A), IgG2a (Figure 7B), IgG1, IgG2a, IgG2b, and IgG3 (Figure 7C), and total IgG (Figure 7D), epitope profiles for different immunogens showed similar trends with SARS-2 RBD-eVLP clustering farthest from the four dual quartet immunogens and the dual quartet immunogens showing more overlap with each other. Dual quartet RBD-mRNA mapped closer to SARS-2 RBD-eVLP in all cases (Figures 7A-7E), as is especially apparent for IgG2a (Figure 7B), total IgG (Figure 7D), and the FcγRs (Figure 7E). By contrast, dual quartet RBD-EABR-mRNA clustered further away from SARS-2 RBD-eVLP and closer to dual quartet RBD-eVLP. Dual quartet RBD-NP mapped similarly to dual quartet RBD-EABR-mRNA. To further assess differences in epitope recognition between different immunogens, a three-dimensional UMAP plot was generated using all data (Figure.7F). Trends in the 3D map were consistent with the 2D UMAPs in that responses to the dual quartet immunogens were distinct from responses to SARS-2 RBD-eVLP, and responses to dual quartet RBD-EABR-mRNA tended to map between dual quartet RBD-mRNA and the protein-based dual quartets, consistent with the EABR approach that combines traditional mRNA- and protein-based vaccinations.^16^ In addition, the finding that SySPEM scores for dual quartet RBD-mRNA overlapped more with scores for SARS-2 RBD-eVLP than with scores for the other dual quartet immunogens is consistent with more class 1/class 2 RBD epitope targeting by dual quartet RBD-mRNA and SARS-2 RBD-eVLP (Figure S6).

Overall, the SySPEM studies confirmed targeting of more conserved RBD epitopes by the mRNA-based dual quartet immunogens, particularly the EABR-modified version, indicating successful conversion of the desirable properties of protein-based mosaic-8 immunogens. With detailed analyses, however, we found distinct epitope profiles for IgG subclass and FcγR-binding IgGs: i.e., the same antigens presented via different modalities (mRNA plus and minus EABR sequences and protein antigens on NPs or eVLPs) showed divergent SySPEM epitope profiles. In addition, SySPEM profiling of binding data rationalized differences in neutralization profiles when comparing two similar immunogens presented differently (e.g., dual quartet RBD-EABR-mRNA versus dual quartet RBD-mRNA).

## DISCUSSION

mRNA-based COVID-19 vaccines have been critical for preventing severe disease and death after SARS-2 infection,^55^ thereby helping keep the worldwide pandemic in check. A recent modification of Moderna’s mRNA-1273 vaccine involved changing the encoded antigen from a SARS-2 spike trimer to a membrane-bound spike N-terminal domain and RBD.^56^ The new vaccine, mRNA-1283, exhibited equivalent or superior induced immune responses compared to mRNA-1273 in mice^56^ and humans,^57^ thus supporting further development of RBD-based COVID-19 and broader CoV vaccines.

We previously described protein-based RBD-NP immunogens that show promise as a pan-sarbecovirus vaccine.^7-10,12,26,27^ However, manufacturing a protein-based mosaic-8 NP vaccine presents challenges: mosaic-8 RBD-NP requires expression and purification of nine protein components (eight RBDs and an NP) to generate a conjugated RBD-NP, which then requires additional purification and characterization. mRNA vaccines offer the possibility of simpler manufacturing,^58^ but developing eight individual RBD constructs would also be challenging, especially since mRNA delivery might not achieve co-display of all eight RBDs on cell surfaces and/or eVLPs.

Here, we report an efficient approach to develop an mRNA-encoded version of a broadly cross-reactive mosaic-8 RBD-NP vaccine. This approach, inspired by protein-based dual quartet RBD-NPs,^12^ involves genetically encoding sarbecovirus RBDs arranged as two membrane-bound quartets, each comprising four tandemly arranged RBD ectodomains. RBD quartets offer the opportunity for optimal Ab induction by displaying a greater number of RBDs per transmembrane region compared with pre-sentation of individual RBDs. The mRNA immunogens described here display dual RBD quartets at cell surfaces (dual quartet RBD-mRNA) or at both the surfaces of cells and secreted eVLPs (dual quartet RBD-EABR-mRNA) when an EABR cytoplasmic domain sequence is included.^16^ Our analyses showed that dual quartet mRNA immunogens elicited equivalent or superior Ab responses, improved FcγR binding of elicited Abs, and increased T cell responses compared with protein-based dual quartet RBD-NPs. In addition, the dual quartet mRNA immunogens induced balanced T_H_1/T_H_2 responses, thereby stimulating both cellular and humoral immune responses. Finally, of critical importance for a potential pan-sarbecovirus vaccine, DMS showed that mRNA-based dual RBD quartet immunogens induced Abs against conserved RBD epitopes, as required for a pan-sarbecovirus vaccine and as observed for protein-based mosaic-8 RBD-NP immunogens.^7-10,12,26,27^

Despite eliciting high Ab binding titers against both SARS-2 variants and non-SARS-2 sarbecoviruses, neutralization titers for mosaic-8 immunogens (dual quartet RBD mRNAs, dual quartet RBD-NPs, and dual quartet RBD-eVLPs) were lower against SARS-2 variants than against zoonotic sarbecoviruses (Figures 2C and S2B). However, we recently reported increased neutralizing titers against hard-to-neutralize viruses, including Omicron, elicited by pulsatile release of a single injection of mosaic-8 RBD-NPs using atomic layer deposition (ALD) compared with bolus prime and boost injections of the same immunogen.^13^ ALD is also an effective delivery mechanism for mRNA vaccines,^59^ offering the possibility that increased neutralizing potencies would be observed for ALD formulations of mRNA-based mosaic-8 immunogens. In addition, improved neutralization of SARS-2 variants might be achieved by changing the RBD composition of mosaic-8 immunogens; e.g., a computationally designed mosaic-7 RBD-NP elicited stronger neutralizing and binding Ab responses than original mosaic-8 RBD-NPs,^10^ and in independent studies of the immunogenicity of different mosaic RBD immunogen compositions, we observed increased cross-reactive responses when we removed the SARS-2 RBD from mosaic RBD immunogens.^9,10^ Thus, altering the composition of dual RBD quartets (e.g., adding an Omicron RBD to an RBD quartet, adding clade 3 RBDs, and/or removing all SARS-2 RBDs) might improve neutralization potencies against SARS-2 variants.

In the absence of neutralization, binding Ab responses can trigger immune responses to clear infected cells through Fc-mediated effector functions^60^ and/or complement activation,^61^ and binding Ab titers correlate with protection in humans and animals vaccinated with COVID-19 mRNA vaccines.^28-30^ Indeed, human mAbs that neutralized early SARS-2 strains but lost neutralizing activity against SARS-2 Omicron variants retained Fc effector functions^60^ and non-neutralizing human anti-RBD mAbs protected from a SARS-2 challenge in animal models.^62^ In addition, T cell responses conferred protection from an Omicron challenge in the absence of neutralizing Ab responses,^63^ and CD4^+^ T cells producing IFN-γ play critical roles in controlling SARS-CoV-2 infection independently of Abs.^64^ Thus, even in the absence of strong neutralizing Ab titers against SARS-2 variants, the ability of mosaic-8 mRNA immunogens to elicit Abs that exhibit broad binding across sarbecovirus RBDs, as well as robust and balanced T cell responses, are desirable traits for a protective pan-sarbecovirus vaccine.

For characterizing dual quartet immunogens, we describe a systems serology technique, SySPEM, that quantifies the degree of binding to individual epitopes on an antigen across IgG subclasses and FcγR-binding IgGs, therefore allowing comparisons of epitope profiles elicited by different immunogens that are not possible using traditional systems serology.^51^ SySPEM allows higher throughput epitope mapping than, e.g., EMPEM,^53^ while still delivering some of the same information and also delineating epitopes recognized by different Ab classes. In contrast to epitope mapping competition ELISAs,^65^ multiplexed SySPEM analyses use minimal sample volumes, do not rely on a mAb panel of known epitopes, and provide more precise epitope definitions based on the locations of introduced N-glycans. SySPEM and DMS results are complementary, but SySPEM experiments are easier to execute, require less material, and can allow quantitative comparisons between responses to distinct epitopes for different immunogens and IgG classes. Also, the SySPEM results reported here yielded mechanistic insights that were not revealed by DMS (e.g., quantifying differences in responses to dual quartet RBD-mRNA and dual quartet RBD-EABR-mRNA). In addition, we used SySPEM to show that the same antigen (dual quartet RBDs) delivered through mRNA- or protein-based vaccine modalities elicited distinct IgG class-specific epitope profiles, suggesting that the way an antigen is presented can impact its epitope recognition by the immune system.

SySPEM revealed potential advantages of the mRNA-EABR approach compared with a traditional mRNA immunogen; e.g., although overall binding and neutralizing Ab titers were similar, the dual quartet RBD-EABR-mRNA immunogen elicited stronger targeting of conserved class 4 and class 5 epitopes, including against the difficult to neutralize SARS-2 XBB.1.5 (as revealed by DMS), and weaker targeting of variable class 1/class 2 epitopes than dual quartet RBD-mRNA, suggesting that the addition of an EABR sequence could improve the effectiveness of an mRNA-encoded dual quartet RBD pan-sarbecovirus vaccine. For example, immunogen presentation on circulating eVLPs would promote wider antigen distribution to potentially engage more B cells.^16,66^ Indeed, circulating RBD-eVLPs resulting from a dual quartet RBD-EABR-mRNA immunogen offer similar advantages to a recently described mRNA-delivered RBD protein NP^67^ but also encode membrane-bound antigens at cell surfaces that could trigger additional immune responses. eVLP budding in the EABR approach might facilitate continuous replenishment of the cell surface with antigen, potentially increasing overall levels of antigen being displayed. The small size of eVLPs compared with a cell also facilitates efficient internalization by antigen-presenting cells,^68-71^ which could result in enhanced antigenic peptide presentation by class II MHC and activation of T follicular helper cells to promote B cell activation and germinal center formation.^66^ Improvements for mRNA vaccines by including EABR appear to be general, as the EABR approach enhances vaccine-induced humoral responses against SARS-2,^7-10,12^ RSV,^22^ and influenza.^21^ We also showed that a bivalent Wuhan-Hu-1/BA.5 spike-EABR-mRNA booster elicited more balanced targeting of multiple RBD epitopes in pre-vaccinated mice than conventional monovalent and bivalent spike-mRNA boosters, which primarily targeted variable RBD epitopes.^66^ Taken together, our findings suggest that EABR-mRNA delivery of mosaic and multi-valent immunogens improves targeting of conserved epitopes to enhance Ab breadth.

Pan-sarbecovirus vaccine candidates evaluated in mice would ideally elicit broad-binding IgG2a and IgG2b subclasses that promote robust Fc effector functions^47,48^ to target conserved epitopes and elicit IgGs that bind to activating Fc receptors (FcγR3 and FcγR4). Using SySPEM, we showed that dual RBD quartet immunogens, including the two mRNA-encoded versions, fulfill these criteria. There are many future potential applications for this new technology for design and evaluation of vaccines against a variety of viruses and other pathogens. For example, studies to establish correlates of protection of vaccines could include SySPEM evaluations to determine relative protective effects of epitope targeting by different classes of IgGs, thus establishing desirable parameters for future vaccine efforts. In addition, SySPEM can be used to determine whether some epitopes elicit IgGs with different potential Fc effector functions to inform immunogen design for eliciting optimal immune responses against a particular pathogen.

In summary, our data support advancement of dual quartet RBD-mRNA immunogens with an optimal RBD composition into clinical development as a next-generation booster vaccine to provide protection from emerging SARS-2 variants and to reduce the chances that a future zoonotic sarbecovirus spillover will cause a new epidemic or pandemic.

### Limitations of the study

Because it is not possible to find analogous doses for mRNA-encoded and protein-encoded vaccines, it is difficult to interpret differences in elicited Ab quantities and neutralization potencies when comparing the effects of immunizing animals with mRNA-LNP versus protein-based immunogens. In addition, our assays to compare immune responses included serum Ab binding and neutralization, IgG subclass and FcγR-binding profiling, and T cell analyses but not protection assessment in a challenge model. Challenge studies would be required once we settle on the optimal RBD quartet composition prior to a clinical trial. Relevant to the present study, we previously demonstrated mosaic-8 protection from matched and mismatched challenges in non-human primates and in K18-hACE2 transgenic mice, even for an immunogen (mosaic-8gm) that induced only weakly neutralizing Ab titers,^8^ and the neutralizing titers for mRNA-based dual quartet immunogens reported here are equivalent or superior to those that conferred protection in our previous paper^8^ and in results reported by others.^62,63^ Finally, upon identifying the optimal RBD quartet composition, peptide pools derived from all eight RBDs in the quartets can be used in addition to the SARS-2 spike-derived peptide pool that was used for the T cell assays in this study.

## STAR★METHODS

### EXPERIMENTAL MODEL AND STUDY PARTICIPANT DETAILS

#### Mice

Mouse procedures were approved under ACUA 0083-24 by the Labcorp Institutional Animal Care and Use Committee, and all experiments conform to relevant regulatory standards. Six-to 7-week-old female BALB/c mice from Charles River Laboratories (RRID: IMSR_JAX:000664) were housed at Labcorp Drug Development, Denver, PA for immunizations. All animals were drug and test naive and healthy upon receipt. The animals were monitored during a 7-day acclimation period, then randomly assigned to experimental groups of 10 animals. Animals weighed between 15.2 and 19.9 grams at the start of the experiment, with an average weight of 18.3 grams. Mouse cages were kept in a climate-controlled room (68°C–79°C) at 50 ± 20% relative humidity and provided with Rodent Diet #5001 (Purina Lab Diet) *ad libitum*.

#### Cell lines

HEK293T cells (RRID:CVCL_0063) for flow cytometry experiments and pseudovirus production were cultured in Dulbecco’s modified Eagle’s medium (DMEM, Gibco) supplemented with 10% heat-inactivated fetal bovine serum (FBS, Bio-Techne), 1% Penicillin/Streptomycin (Gibco), and 1% L-Glutamine (Gibco) at 37°C and 5% CO_2_. HEK293T-hACE2 cells (RRID:CVCL_A7UK)^41^ and high-hACE2 HEK-293T cells (kind gift of Kenneth Matreyek, Case Western Reserve University) for neutralization assays were cultured in DMEM (Gibco) supplemented with 10% heat-inactivated FBS (Bio-Techne), 5 mg/mL gentamicin (Sigma-Aldrich), and 5 mg/mL blasticidin (Gibco) at 37°C and 5% CO_2_.

Expi293F cells (RRID:CVCL_D615, ThermoFisher) for protein expression were maintained at 37°C and 8% CO_2_ with 130 rpm shaking in Expi293 expression medium (ThermoFisher).

293T-based cell lines were derived from female donors and not specially authenticated or tested for mycoplasma contamination.

#### Microbes

BL21 (DE3) *E. coli* (Agilent) were cultured in LB Miller’s formulation broth supplemented with 0.8% (w/v) glucose and appropriate antibiotic for expression of SpyCatcher003-mi3 nanoparticles. Cultures were grown at 37°C with 200 rpm shaking until induction, at which time the temperature was decreased to 22°C.

Saccharomyces cerevisiae strain AWY101 was cultured at 30°C with 275 rpm shaking in selective medium containing 6.7g/L Yeast Nitrogen Base, 5.0 g/L Casamino acids, 1.065 g/L MES acid, and 2% w/v dextrose. RBD expression in RBD library-transformed yeast was induced in selective medium containing galactose (6.7g/L Yeast Nitrogen Base, 5.0 g/L Casamino acids, 1.065 g/L MES acid, and 2% w/v galactose plus 0.1% w/v dextrose).

### METHOD DETAILS

#### Membrane-bound RBD quartet constructs

Sequences of the extracellular domains of RBD quartets 4a and 4b were based on previously reported SpyTag-Quartet (GenBank PP13603) and SpyTag-Alternate Quartet (GenBank PP136032, Addgene plasmid ID 214728) sequences.^12^ mRNA constructs encoded a mouse Ig heavy chain signal peptide, codon-optimized RBD quartets 4a and 4b in which individual RBDs were separated by nine-residue Gly/Ser/Thr linkers followed by a five-residue Gly/Ser linker, the SARS-1 spike transmembrane domain, and a truncated spike cytoplasmic tail^80^ (GenBank AAP13441.1, residues [1218–1235]). For RBD quartet 4a-EABR and 4b-EABR constructs, an endocytosis-preventing motif (EPM),^16^ four-residue Gly/Ser linker, and the EABR^16^ sequence were appended to the C terminus. For *in vitro* assays, these constructs were cloned into the IVTpro T7 plasmid (Takara 6144), containing the immunogen open reading frame flanked by 5′ and 3′ untranslated regions (UTRs) and a genetically encoded poly(A) tail.

#### mRNA synthesis

For *in vitro* assays, IVTpro T7 plasmids encoding codon-optimized RBD quartet 4a, RBD quartet 4b, RBD quartet 4a-EABR, and RBD quartet 4b-EABR constructs were linearized by BspQI restriction digestion (NEB, R0712) and subsequently purified using the Zymo DNA Clean & Concentrator-25 kit (Zymo, D4034). mRNA was generated by *in vitro* transcription using the IVTpro T7 mRNA Synthesis Kit (Takara, 6144) following manufacturer’s instructions, with the addition of the CleanCap Reagent AG (3′ OMe) (TriLink, *N*-7413) and complete substitution of uridine with N1-Methylpseudouridine-5′-Triphosphate (TriLink, *N*-108) to reduce immunogenicity.^16^ mRNA was then purified by lithium chloride precipitation and an ethanol wash following standard protocols.^81^ Purified mRNA was resuspended in nuclease-free water and stored at −80°C.

For mRNAs used for *in vivo* immunization studies, constructs were synthesized by RNAcore (https://www.houstonmethodist.org/research-cores/rnacore/) using proprietary manufacturing protocols including incorporation of the CleanCap Reagent AG (3′ OMe) (TriLink, *N*-7413) and complete substitution of uridine with N1-Methylpseudouridine-5′-Triphosphate (TriLink, *N*-108). mRNAs were purified by oligo-dT affinity purification. To remove double-stranded RNA contaminants, mRNAs were further purified using a cellulose-based method as described.^82^ In brief, cellulose fibers (0.2 g/mL) were equilibrated in chromatography buffer (10mM HEPES pH 7.2, 0.1 mM EDTA, 125 mM NaCl, 16% ethanol) and added to microcentrifuge spin columns. mRNA diluted in chromatography buffer was added and shaken vigorously at room temperature for 30 min. The flow-through containing single-stranded mRNA was collected and purified with a sodium acetate precipitation and an ethanol wash. The final purified mRNA was filtered through a 0.2 μm filter before storage at −80°C.^16,82^

#### mRNA transfections and flow cytometry

HEK293T cells were seeded at 1 × 10^6^ cells per well in 6-well plates and incubated for 20 h before transfection with 2 μg of mRNA encoding RBD quartet 4a, RBD quartet 4b, and/or their EABR-fused counterparts (4a-EABR, 4b-EABR) using Lipofectamine MessengerMax (Thermo Fisher Scientific). Supernatants were collected 48 h post-transfection for eVLP purification by ultracentrifugation on a 20% sucrose cushion as described.^16^ Cells were gently detached, resuspended in PBS+ (PBS +2% FBS), and 100 μL aliquots were taken for flow cytometry.

For the experiment shown in Figure S1A, cells were stained with the cross-reactive anti-RBD mAb C118^18,19^ (2.5 μg/mL) for 30 min in the dark at room temperature, washed, and subsequently stained with Alexa Fluor 647-conjugated anti-human IgG secondary Ab (Invitrogen, A21445; 1:4,000) for 15 min. After a final wash, cells were resuspended in PBS+ and analyzed on an Attune NxT Flow Cytometer (Thermo Fisher).

For the experiment shown in Figure S1C, cells were either singly transfected or co-transfected with combinations of RBD quartet 4a and 4b constructs and stained simultaneously with 2.5 μg/mL of Alexa Fluor 647-conjugated anti-Rs4081 RBD^26^ and Alexa Fluor 488-conjugated M8a-7 IgG (anti-WIV1 RBD).^27^ mAbs for 30 min at room temperature in the dark. After washing and resuspension, samples were analyzed on an Attune NxT Flow Cytometer. Results were plotted using FlowJo 10.5.3 software.

#### Production of eVLPs

Dual quartet RBD-eVLP and SARS-2 RBD-eVLP samples were generated as described^16^ via transient transfection of Expi293F cells (ThermoFisher). To generate dual quartet RBD-eVLPs, DNA plasmids encoding RBD quartet 4a-EABR and RBD quartet 4b-EABR constructs were mixed at a ratio of 1:1. 72 h post-transfection, cells were centrifuged at 400 x g for 10 min, supernatants were passed through a 0.45 μm syringe filter and concentrated using Amicon Ultra-15 centrifugal filters with 100 kDa molecular weight cut-off (Millipore). eVLPs were purified by ultracentrifugation at 50,000 rpm (135,000 x g) for 2 h at 4°C using a TLA100.3 rotor and an Optima TLX ultracentrifuge (Beckman Coulter) on a 20% w/v sucrose cushion. After removal of supernatants by aspiration, pellets were resuspended in 200 μL sterile PBS at 4°C overnight. To remove residual debris, samples were centrifuged at 10,000 x g for 10 min, and supernatants were collected. eVLPs were further purified by SEC using a Superose 6 Increase 10/300 column (Cytiva) equilibrated with PBS. Peak fractions corresponding to eVLPs were combined and concentrated to 250–500 μL in Amicon Ultra-4 centrifugal filters with 100 kDa molecular weight cut-off. Samples were aliquoted and stored at −20°C.

The amount of SARS-2 RBD on SARS-2 RBD-eVLPs and the amount of dual quartet RBD on dual quartet RBD-eVLPs were determined by quantitative Western blot analysis. Various dilutions of SEC-purified eVLP samples and known amounts of either soluble SARS-2 RBD protein (Sino Biological, 40591-V08H-B-20) or purified soluble SpyTagged dual quartet RBDs (SpyTagged RBD quartets 4a and 4b were mixed at a ratio of 1:1) were separated by SDS-PAGE and transferred to nitrocellulose membranes (0.45 μm) (Thermo Fisher Scientific, LC2001). Rabbit anti-SARS-2 S1 polyclonal IgG (Thermo Fisher Scientific, PA5-116916; 1:1,000) and HRP-conjugated goat anti-rabbit IgG (Abcam, ab98467; 1:10,000) were used. Protein bands were visualized using ECL Prime Western Blotting Detection Reagent (Cytiva, RPN2232). Band intensities of the respective standards and eVLP sample dilutions were measured using ImageJ to determine RBD amounts.

#### LNP encapsulation of mRNAs

Purified dual quartet RBD-encoding mRNAs (RBD quartet 4a + RBD quartet 4b or RBD quartet 4a-EABR + RBD quartet 4b-EABR; 1:1 ratio) for *in vivo* studies were formulated in LNPs as previously described.^83^ 1,2-distearoyl-*sn*-glycero-3-phosphocholine, cholesterol, a PEG lipid, and an ionizable cationic lipid dissolved in ethanol were rapidly mixed with an aqueous acidic solution containing mRNA using an in-line mixer. The ionizable lipid and LNP composition are described in the international patent application WO2017075531(2017). The post in-line solution was dialyzed with PBS to remove the ethanol and displace the acidic solution. LNP was measured for size (60–65 nm) and polydispersity (PDI <0.075) by dynamic light scattering (Malvern Nano ZS Zetasizer). Encapsulation efficiencies were >97% as measured by the Quant-iT Ribogreen Assay (Invitrogen).

#### Protein expression

RBDs, RBD quartets, and soluble sarbecovirus spike-6P^78^ (SARS-2 WA1 and JN.1) or spike-2P (XBB.1.5, SARS-1, Rf1, Rs4091, Yun11, BM48-31, BtKY72) trimers were expressed as described.^8,12,13^ Briefly, AviTag and/or His-tagged proteins were purified from transiently-transfected Expi293F cells (ThermoFisher) by nickel affinity chromatography (HisTrap HP, Cytiva) and SEC (Superose 6 Increase 10/300, Cytiva).^8,42,84^ Fractions corresponding to proteins of interest were pooled, concentrated, and stored at 4°C. Biotinylated proteins were generated as described^85^ by co-transfection of AviTag/His-tagged spike and RBD constructs with a plasmid encoding an endoplasmic reticulum-directed BirA enzyme (kind gift from Michael Anaya, Caltech). The anti-Rs4081, anti-RmYN02, and anti-WIV1 RBD mAb IgGs from Figure S1 and the mAb IgGs and human ACE2-Fc protein shown in Figure S9C were expressed, purified, and classified as recognizing the RBD epitopes as described.^19,26,27,42^

#### Preparation of RBD-NPs

For making dual quartet RBD-NPs, SpyCatcher003-mi3–C-Tag (Addgene 159995) subunits were expressed in BL21 (DE3) *E. coli* (Agilent) as described.^86^ SpyCatcher003-mi3–C-Tag bacterial cell pellets were resuspended in 20 mL of 20 mM Tris-HCl pH 8.5, 300 mM NaCl, supplemented with 0.1 mg/mL lysozyme, 1 mg/mL cOmplete mini EDTA-free protease inhibitor (Roche), and 1.0 mM PMSF. After incubation at 4°C for 45 min with end-over-end mixing, an Ultrasonic Processor equipped with a microtip (Cole-Parmer) was used to perform sonication on ice (four times for 60 s, 50% duty-cycle), and cell debris were cleared by centrifugation (35,000 x g for 45 min at 4°C). SpyCatcher003-mi3–C-Tag was purified by ammonium sulfate precipitation (170 mg per mL of lysate) and resuspension, followed by SEC using a HiPrep Sephacryl S-400 HR 16–60 column (GE Healthcare) equilibrated with PBS as described.^86^ Dual quartet RBD-NPs were generated by incubating purified SpyCatcher003-mi3–C-Tag with a 1:1 molar ratio of purified SpyTagged RBD quartets at 4°C in PBS.

#### Immunization of mice

Mice were immunized by intramuscular injection on days 0 and 28 with 1.5 or 0.5 μg dual quartet RBD-mRNA, 1.5 or 0.5 μg dual quartet RBD-EABR-mRNA, 5 μg dual quartet RBD-NP, 5 μg dual quartet RBD-eVLP, or 5 μg SARS-2 RBD-eVLP (based on RBD content for protein-based immunogens). Prior to immunizations, dual quartet RBD-NP, dual quartet RBD-eVLP, and SARS-2 RBD-eVLP immunogens were mixed with Addavax adjuvant (InvivoGen; 50% v/v). Serum samples for ELISAs and neutralization assays were obtained on indicated days by retro-orbital bleeding.

#### ELISAs

For eVLP sandwich ELISAs, 96-well high-binding plates (Corning, #9018) were coated overnight at 4°C with capture mAb (5 μg/mL in 0.1 M NaHCO_3_, pH 9.8). Plates were blocked with 3% BSA and 0.1% Tween 20 in TBS (TBS-T) for 30 min at room temperature, followed by addition of serially diluted purified eVLP samples for 2 h. After washing, biotinylated detection Abs (5 μg/mL in blocking buffer) were added for 2 h at room temperature. Plates were washed three times with TBS-T, incubated with streptavidin-HRP (Ab-cam, 7403; 1:20,000) for 30 min, washed again, and developed with 1-Step Ultra TMB-ELISA substrate solution (ThermoFisher). Reactions were stopped with 1 N HCl and absorbance was measured at 450 nm.

For the ELISA in Figure S1B, plates were coated with either anti-Rs4081^26^ or anti-RmYN02.^26^ mAbs and detected with biotinylated C118.^18,19^ For the ELISA in Figure S1D, plates were coated with anti-WIV1^27^ and detected with biotinylated anti-Rs4081.^26^

For serum ELISAs, purified His-tagged RBD (2.5 μg/mL in 0.1 M sodium bicarbonate buffer pH 9.8) was coated onto Nunc MaxiSorp 384-wellplates (Sigma) and incubated overnight at 4°C. After blocking with 3% bovine serum albumin (BSA), 0.1% Tween 20 in Tris-buffered saline (TBS-T) for 1 h at room temperature, blocking solution was removed by aspiration. For serum ELISAs shown in Figure 2 and Figure S2, mouse serum (1:100 initial dilution) was serially diluted by 3.1-fold in TBS-T/3% BSA and then added to plates for 3 h at room temperature, followed by washing with TBS-T. Plates were then incubated for 1 h with a 1:100,000 dilution of HRP-conjugated anti-IgG secondary (goat anti-mouse IgG; Abcam; RRID: AB_955439), and plates were washed with TBS-T. For mAb ELISAs shown in Figure S9, purified mAbs were serially diluted by 3.1-fold in TBS-T/3% BSA and then added to plates for 3 h at room temperature, followed by washing with TBS-T. Plates were then incubated for 1 h with a 1:100,000 dilution of HRP-conjugated anti-IgG secondary (goat anti-human IgG; SouthernBiotech 2014-05), and plates were washed with TBS-T. SuperSignal ELISA Femto Substrate (ThermoFisher) was added as per manufacturer’s instructions, and luminescence was read at 425 nm. Data were collected in duplicate and midpoint titers (ED_50_ values) were obtained using Graphpad Prism 10.5.0 assuming a four-parameter dose-response curve fit.

#### Pseudovirus neutralization assays

Lentiviral-based viruses were prepared as described in HEK293T cells^18,87^ using genes encoding spike sequences lacking C-terminal residues in the cytoplasmic tail: deletions of 21 residues (SARS-2 variants) or 19 residues (SARS-1, Khosta-2-SARS-1 chimera, BtKY72-SARS-1 chimera). BtKY72 (containing K493Y/T498W substitutions) and Khosta-2 pseudoviruses were made with chimeric spikes in which the RBD from SARS-1 (residues 323–501) was substituted with the RBD from BtKY72 K493Y/T498W (residues 327–503) or Khosta-2 (residues 324–500) as described.^88^ Cells were co-transfected with HIV-1-based lentiviral plasmids, a luciferase reporter gene, and a coronavirus spike construct, resulting in lentivirus-based pseudovirions expressing a sarbecovirus spike protein. Supernatants were harvested 48–72 h post-transfection, filtered, and stored at −80°C. Pseudovirus infectivity was determined by titration using HEK293T-hACE2 cells.

Prior to neutralization assays, serum from immunized mice was heat-inactivated at 56°C for 10 min. Heat-inactivated serum was 3-fold serially diluted and incubated with pseudovirus for 1 h at 37°C, then the serum/virus mixture was added to HEK293T-hACE2 target cells or high-hACE2 HEK-293T cell line expressing hACE2 encoded with a consensus Kozak sequence (for SHC014 assays; kindly provided by Kenneth Matreyek, Case Western Reserve University) and incubated for 48 h at 37°C. After removing media, cells were lysed with Britelite Plus reagent (Revvity Health Sciences), and luciferase activity was measured as relative luminesce units (RLUs). Relative RLUs were normalized to RLUs from cells infected with pseudotyped virus in the absence of antiserum. Each sample was tested in duplicate and average half-maximal inhibitory dilutions (ID_50_ values) were derived in AntibodyDatabase^79^ using 4-parameter nonlinear regression.

#### ELISpot assays

Mice were euthanized on day 77 or 78, and spleens were collected and processed as described.^16^ Briefly, spleens were homogenized using a gentleMACS Octo Dissociator (Miltenyi Biotec). Cells were passed through a 70 μm tissue screen, centrifuged at 1,500 rpm for 10 min, and resuspended in CTL-Test media (ImmunoSpot) containing 1% GlutaMAX supplement (Gibco). ELISpot assays were performed in duplicate as described.^16^ A SARS-2 (Wuhan-1) spike PepMix pool of 315 peptides (15-mers with 11 amino acid overlap; JPT Peptide Technologies) was added to mouse IFNγ/IL4 double-color ELISpot plates (ImmunoSpot) at a concentration of 2 μg/mL, and 300,000 splenocytes were added per well. Plates were incubated at 37°C for 24 h. Biotinylated detection reagents, streptavidin-alkaline phosphatase (AP), and substrate reagents were added according to the manufacturer’s guidelines. To stop the reactions, plates were gently rinsed with water three times. Plates were air-dried for two hours in a running laminar flow hood. Numbers of spots were quantified using a CTL ImmunoSpot S6 Universal-V Analyzer (Immunospot).

#### DMS epitope mapping

DMS experiments to map epitopes recognized by serum Abs were performed in biological duplicates using independent mutant RBD yeast libraries (WA1,^83^ XBB.1.5,^84^ and SARS-1^77^ generously provided by Tyler Starr, University of Utah) as described.^8,39^ Sera that had been heat inactivated for 30 min at 56°C were incubated twice with 50 OD units of AWY101 yeast transformed with an empty vector in order to remove non-specific yeast-binding Abs. Expression of RBDs in libraries was induced in galactose-containing synthetic defined medium with Casamino acids (6.7g/L Yeast Nitrogen Base, 5.0 g/L Casamino acids, 1.065 g/L MES acid, and 2% w/v galactose plus 0.1% w/v dextrose). After 18 h of induction, cells were washed twice and incubated with serum under conditions of gentle agitation for 1 h at room temperature. Cells were labeled for 1 h with a secondary Ab (1:200 Alexa Fluor-647-conjugated goat anti-mouse-IgG Fc-gamma, Jackson ImmunoResearch 115-605-008, RRID:AB_2338904) after washing twice.

Stained yeast cells were examined by fluorescence-activated cell sorting (FACS) using a Sony SH800 cell sorter. Cells were gated to capture RBD mutants that showed reduced Ab binding compared to a control. Cells were collected for each sample until ~5 x 10^6^ RBD^+^ cells were processed, which corresponds to ~5 x 10^5^- 1 x 10^6^ RBD^+^ Ab-escaped cells. These cells were grown overnight in synthetic defined media (6.7 g/L Yeast Nitrogen Base, 5.0 g/L Casamino acids, 1.065 g/L MES acid, and 2% w/v dextrose, 100 U/mL penicillin, 100 μg/mL streptomycin) to expand cells prior to plasmid extraction. DNA extraction and Illumina sequencing were done as described^12^ (raw sequencing data available on NCBI SRA Bioproject: PRJNA1067836 and Biosamples: SAMN52937385). Escape fractions were computed as described^12,39^ using Swift DMS^12^ (available upon request). Escape scores were calculated using a filter to remove variants with mutations that escaped binding because of poor expression, >1 amino acid mutation, or low sequencing counts.^12,89^

Escape map visualizations (static line plots, logo plots, and structural depictions) were created using Swift DMS.^12^ Line heights show the escape score for a particular amino acid substitution, as described.^12^ In some visualizations, sites were categorized based on RBD epitope region^19,42-44^: class 1 (pink; RBD residues 403, 405, 406, 417, 420, 421, 453, 455–460, 473–478, 486, 487, 489, 503, 504); class 2 (purple; residues 472, 479, 483–485, 490–495), class 3 (blue; residues 341, 345, 346, 437–450, 496, 498–501), class 4 (orange; residues 365–390, 408), class 5 (green; residues 352–357, 396, 462–468). In structural depictions of DMS results, an RBD surface (PDB 6M0J) was colored by the site-wise escape metric at each site, with red scaled at an escape fraction of 2.0. Residues exhibiting the highest escape fractions were labeled with their residue number, which was colored according to epitope class. Logo plot residues are colored according to RBD epitopes within different classes as indicated on the legend.

We stratified DMS escape fraction values into four groups. Escape fractions for each RBD substitution range from 0 (no cells with this substitution were sorted into the escape bin) to 1 (all cells with this substitution were sorted into the serum Ab escape bin).^38^ The sum of escape fractions for all substitutions at a specific site is represented by the total escape peak.^38^ DMS profiles with total escape peaks <0.5 at all sites were classified as polyclass responses. DMS profiles with total escape peaks of 0.5–1, >1 to 2, or >2 at one or more sites were classified as weak, moderate, or strong escape profiles, respectively, corresponding to their RBD epitope (Figure S6).

#### Ab subclass and FcγR binding profiling

Serum samples from immunized mice were analyzed in systems serology^51^ experiments as described^13^ using modifications of a Luminex assay to quantify the levels of antigen-specific Ab subclasses and FcγR binding profiles, which allows for simultaneous detection and quantification of multiple analytes (i.e., different antigens) in a single sample.^90^ In brief, avidin (Sigma-Aldrich Catalog #: A9275-25MG) was coupled by carbodiimide-NHS ester chemistry to magnetic Luminex microspheres (LuminexCorp) using Sulfo-NHS (ThermoFisher Catalog number 24510) and 1-Ethyl-3-[3-dimethylaminopropyl]carbodiimide hydrochloride (EDC) (ThermoFisher Catalog number 22980) according to the manufacturer’s instructions. Microspheres were blocked for 30 min with 1% BSA in PBS pH 7.4 (assay buffer) and then washed twice with PBS. Microspheres were incubated with biotinylated antigen (sarbecovirus spike trimer, RBD, or control antigen) at room temperature for 2 h in assay buffer followed by blocking with 10 μM biotin (Millipore Sigma, Catalog B4501-100MG). Antigen-coupled microspheres were combined then incubated with heat-inactivated serum samples for 1 h at room temperature in 96-well plates (Corning) at 1:200 or 1:1000 dilutions. Unbound IgGs were removed by washing twice with 200 μL of assay buffer. Total IgG was detected using a PE-conjugated secondary Ab recognizing mouse IgG (Southern Biotech 1030-09S). For evaluating antigen binding by different IgG subclasses, secondary Abs against mouse IgG subclasses (Southern Biotech 1070-09S, 1080-09S, 1090-09S, 1100-09S; PE-coupled anti-IgG1, IgG2a, IgG2b, IgG3) were added at a 1:1000 dilution in assay buffer and incubated for 1 h with continuous shaking at room temperature. Excess primary and secondary Abs were removed by washing twice with 200 μL of assay buffer. Beads were resuspended in 200 μL of assay buffer, run in duplicate, and flowed in single file past two lasers on a Luminex FLEXMAP 3D Instrument System. In a Luminex assay, each bead region is impregnated with a different ratio of fluorescent dyes such that bead region identity, and thereby antigen identity, is interrogated by the first laser. A second laser interrogates the signal of secondary Ab binding. Median fluorescence intensity (MFI) was calculated for all samples from duplicate measurements.

For evaluating FcγR-binding IgGs, soluble ectodomains of 6xHis-tagged FcγR2b, FcγR3, and FcγR4 were prepared as described^13^ and biotinylated by co-expression with BirA enzyme in Expi293T cells.^85^ Biotinylated FcγRs were purified on a HisTrap column (VWR) according to the manufacturer’s instructions followed by SEC and then bound to PE-streptavidin (eBioscience). Labeled FcγRs were diluted 1:200 in assay buffer and incubated with serum-coated microspheres for 1 h at room temperature with continuous shaking. Unbound primary and PE-labeled FcγR were removed as described for anti-IgG subclass secondary Abs. Beads were resuspended and run on a Luminex FLEXMAP 3D Instrument System to derive MFI values as described above.

Log_10_-transformed heatmaps of antigen-specific IgG responses were generated using Python (v3.9.16) with the Pandas (v2.0.3), NumPy (v1.23.5), Matplotlib (v3.7.1), and Seaborn (v0.12.2) packages. A soluble influenza hemagglutinin trimer (listed as CA-09 HA in Figure 5; expressed and purified as described^72^) was used as a control to evaluate non-specific binding.

#### SySPEM

One or more potential N-linked glycosylation site sequons (Asn-x-Ser/Thr)^91^ were introduced at solvent-exposed residue(s) within the class 1 and class 2, class 2, class 3, class 4, class 1/4, or class 5 RBD epitopes^19,42-44^ of the WA1 RBD (Figure S9A). Addition of N-glycan(s) was confirmed by SDS-PAGE as a shift in electrophoretic mobility to a higher apparent molecular weight (Figure S9B). Proper folding of RBD KO mutants and expected effects of added N-glycans were verified by assaying the binding of characterized mAbs or a human ACE2-Fc construct^19^ by ELISA (Figure S9C). Binding of serum subsets to RBD wt and RBD KO proteins was measured in duplicate using a Luminex multiplexed bead-based immunoassay as described above. The SySPEM score for each IgG sample/epitope pair was calculated as [1.0 – (RBD KO binding/RBD wt binding) x 100]. SySPEM scores range from limits of 100 (none of the IgGs in the sample bound to the RBD KO; i.e., all of the IgGs recognize the epitope that was targeted by the glycan addition) to 0 (all of the IgGs in the sample bound to the RBD KO; i.e., none of the IgGs recognize the epitope that was targeted by the glycan addition) (Figure S8). Python (v3.9.16) with the Pandas (v2.0.3), NumPy (v1.23.5), Matplotlib (v3.7.1), and Seaborn (v0.12.2) packages were used for box and whisker plots of SySPEM data. For UMAP visualizations in Figure 7, SySPEM scores of each serum sample were organized into a feature matrix where each column contains an epitope class SySPEM score, and each row corresponds to an individual sample. Features were standardized using the standard scalar function on sckit-learn 1.4.2. UMAP was then performed with umap-learn (0.5.9.post2) on the standardized matrix. UMAP parameters: the nearest neighbors (n-neighbors) parameter was set to 12 and minimum distance (min-dist) was set to 0.25 to preserve the best local and global structures on the UMAP plot. Seaborn (v0.12.2) was used to plot resulting UMAPs.

#### Materials, data, and code availability

DMS raw sequencing data is available on the NCBI SRA under BioProject PRJNA1067836, BioSample SAMN52937385. SySPEM scores for each IgG sample/epitope pair will be made publicly available upon publication. Materials are available upon request to corresponding authors with a signed material transfer agreement, and other information required to analyze the data in this paper is available from the lead contacts upon request. Code used for data processing and visualization of DMS, systems serology, and SySPEM results is available upon request. The work is licensed under a Creative Commons Attribution 4.0 International (CC BY 4.0) license, which permits unrestricted use, distribution, and reproduction in any medium, provided the original work is properly cited. To view a copy of this license, visit https://creativecommons.org/licenses/by/4.0/. This license does not apply to figures/photos/artwork or other content included in the article that is credited to a third party; obtain authorization from the rights holder before using such material.

### QUANTIFICATION AND STATISTICAL ANALYSIS

Group sizes, replicates, and statistical analysis details can be found in the main text, figure legends, and method details. We used pairwise comparisons, a method to evaluate sets of mean binding titers against individual viral strains for different immunization cohorts, as described previously^9^ to determine if results from different immunized cohorts were significantly different from each other. For ELISAs and neutralizations (Figure 2), we used analysis of variance (ANOVA) followed by Tukey’s multiple comparison post hoc tests with the Geisser-Greenhouse correction, with pairing by viral strain, of ED_50_s/ID_50_s (converted to log_10_ scale) calculated using GraphPad Prism 10.5.0 to identify statistically significant titer differences between immunized groups for ELISAs. For comparisons of neutralizing titers by strain (Figure S2), statistically significant titer differences between immunized groups for each given strain were determined using unpaired *t* test calculated using GraphPad Prism 11.0.0. For ELISpot assays (Figure 3), statistically significant differences between immunized cohorts were calculated using ordinary one-way ANOVA followed by Tukey’s multiple comparison test using GraphPad Prism. For statistical analysis of systems serology results (Figure 6), responses were aggregated by computing the geometric mean (geomean) across replicate samples for each immunogen-antigen combination. Log_10_-transformed geomeans were compared across immunogen groups using Tukey’s multiple comparison post hoc tests with the Geisser-Greenhouse correction, with pairing by viral strain calculated using GraphPad Prism 11.0.0. Box-and-whisker plots were generated for each IgG subclass or FcγR-binding IgG, displaying individual antigen-level geomeans per immunogen. Python (v3.9.16) with statsmodels (v0.14.4) packages were used for SySPEM Tukey’s HSD posthoc statistical analyses. Statistical significance was annotated in all relevant figures using brackets and asterisk notation (*p* < 0.05 *, *p* < 0.01 **, *p* < 0.001 ***, *p* < 0.0001 ****).

## Supplementary Material

1

Supplemental information can be found online at https://doi.org/10.1016/j.celrep.2026.117335.

## Figures and Tables

**Table:** KEY RESOURCES TABLE

REAGENT or RESOURCE	SOURCE	IDENTIFIER
Antibodies		
C118 IgG	Jette/Cohen et al.^19^	N/A
Anti-Rs4081 IgG (Rs4081 RBD-specific IgG)	Fan/Keeffe et al.^26^	N/A
M8a-7 IgG (WIV1 RBD-specific IgG)	Fan et al.^27^	N/A
Anti-RmYN02 IgG (RmYN02 RBD-specific IgG)	Fan/Keeffe et al.^26^	N/A
Rabbit anti-SARS-2 S1 polyclonal IgG	ThermoFisher	Cat# PA5-116916; RRID:AB_2901546
HRP-conjugated goat anti-rabbit IgG	Abcam	Cat # ab98467; RRID:AB_10674445
Alexa Fluor^®^ 647-conjugated anti-human IgG secondary Ab	Invitrogen	Cat # A21445; RRID:AB_2535862
Goat Anti-Mouse IgG H&L (HRP)	Abcam	Cat #ab6789; RRID: AB_955439
HRP-conjugated goat anti-human IgG Fc	SouthernBiotech	Cat #2014-05; RRID:AB_2795580
Alexa Fluor^®^ 647 AffiniPure^™^ Goat AntiMouse IgG, Fcγ fragment specific	Jackson ImmunoResearch	Cat #115-605-008; RRID: AB_2338904
Total IgG Goat Anti-Mouse IgG, Human ads-PE	Southern Biotech	Cat # 1030-09S; RRID: AB_2794298
Goat Anti-Mouse IgG1, Human ads-PE	Southern Biotech	Cat # 1070-09S; RRID: AB_2794415
Goat Anti-Mouse IgG2a, Human ads-PE	Southern Biotech	Cat # 1080-09S; RRID: AB_2794481
Goat Anti-Mouse IgG2b, Human ads-PE	Southern Biotech	Cat # 1090-09S; RRID: AB_2794524
Goat Anti-Mouse IgG3, Human ads-PE	Southern Biotech	Cat # 1100-09S; RRID: AB_2794577
Bacterial and virus strains		
BL21 CodonPlus (DE3)-RIPL E. coli	Agilent	Cat #230280
SARS-CoV-2 D614G pseudotyped virus	Cohen et al.^7^	N/A
SARS-CoV-2 Omicron BA.5 pseudotyped virus	Cohen et al.^9^	N/A
SARS-CoV-2 Omicron XBB.1.5 pseudotyped virus	Cohen et al.^9^	N/A
SARS-CoV-2 Omicron KP.3 pseudotyped virus	Cohen et al.^9^	N/A
SHC014 pseudotyped virus	Cohen et al.^7^	N/A
WIV1 pseudotyped virus	Cohen et al.^7^	N/A
SARS-CoV-1 (SARS1) pseudotyped virus	Cohen et al.^7^	N/A
Khosta-2 chimera pseudotyped virus	Cohen et al.^8^	N/A
BtKY72 chimera pseudotyped virus	Cohen et al.^8^	N/A
Biological samples		
Immunized mouse serum	This paper	N/A
Chemicals, peptides, and recombinant proteins		
IVTpro^™^ T7 mRNA Synthesis Kit	Takara	Cat #6144
CleanCap Reagent AG (3^′^ OMe)	TriLink	Cat #*N*-7413
N1-Methylpseudouridine-5′-Triphosphate	TriLink	Cat #*N*-108
Lipofectamine^™^ MessengerMax^™^	ThermoFisher	Cat #LMRNA001
Antibody labeling kit (Alexa Fluor 647)	ThermoFisher	Cat #A88068
Antibody labeling kit (Alexa Fluor 488)	ThermoFisher	Cat #A88062
SARS-2 RBD	Sino Biological	Cat #40591-0V08H-B-20
ECL Prime Western Blotting Detection Reagent	Cytiva	Cat # RPN2232
ionizable lipid and LNP composition	Acuitas	Patent Application WO2017075531 (2017)
Quant-iT Ribogreen Assay	Invitrogen	Cat #R11490
Streptavidin-HRP	Abcam	Cat # 7403
Heat-Inactivated Fetal Bovine Serum, Optima	Bio-Techne	Cat #S12450H
Addavax	InvivoGen	Cat # vac-adx-10
Britelite Plus reagent	Revvity Health Sciences, Inc	Cat # 6066769
1-Step^™^ Ultra TMB-ELISA substrate	ThermoFisher	Cat # 34022
SuperSignal ELISA Femto Maximum Sensitivity Substrate	ThermoFisher	Cat # 37074
SARS-2 (Wuhan-1) spike PepMix^™^ pool of 315 peptides	JPT Peptide Technologies	N/A
PE-Streptavidin	eBioscience	Cat # 12-4317-87
Avidin	Sigma-Aldrich	Cat # A9275
Luminex MagPlex-C Microspheres, Various regions	Luminex	Cat # MC100XX
Biotin	Millipore Sigma	Cat #B4501
Soluble HA from A/California/04/09	Cohen et al.^72^	N/A
Spytag-Quartet	Hills et al.^12^	GenBank PP13603
Spytag-Alternate Quartet	Hills et al.^12^	GenBank PP136032
RBD-Avitag-6xHis	Cohen et al.^7,8^	N/A
SpyCatcher003-mi3	Bruun et al.^6^	N/A
Spike-6P-Avitag-6xHis or Spike-2P-Avitag- 6xHis	Cohen et al.^13^	N/A
Soluble mouse FcγR2b	Cohen et al.^13^	Uniprot P08101.2 residues 30-208
Soluble mouse FcγR3	Cohen et al.^13^	Uniprot P08508.1 residues 31-216
Soluble mouse FcγR4	Cohen et al.^13^	Uniprot A0A0B4J1G0 residues 21-204
WA1 RBD with 1 or more potential N-linked glycosylation sites	This paper	N/A
Dual Quartet RBD-mi3 nanoparticle vaccine	Hills et al.^8,12^	N/A
mRNA for *in vivo* immunogenicity studies	This paper	Houston Methodist RNAcore
Critical commercial assays		
Expi293 Expression System Kit	ThermoFisher	Cat # A14635
Deposited data		
Deep mutational scanning sequencing data	This paper; NCBI SRA: Bioproject PRJNA1067836, Biosamples SAMN52937385	N/A
Experimental models: Cell lines		
Expi293F cells	ThermoFisher	RRID:CVCL_D615
HEK293T cells	Pear et al.^73^	Cat # CCLV-RIE 1018; RRID: CVCL_0063
HEK293T-hACE2	BEI	Cat # NR-52511; RRID:CVCL_A7UK
HEK-293T cells expressing high levels of hACE2 (consensus Kozak)	Kenneth Matreyek, Case Western Reserve University	N/A
AWY101 yeast	Wentz and Shusta^74^	N/A
WA1 RBD DMS yeast library	Starr et al.^75^	N/A
XBB.1.5 RBD DMS yeast library	Taylor et al.^76^	N/A
SARS-1 RBD DMS yeast library	Lee et al.^77^	N/A
Experimental models: Organisms/strains		
Female BALB/c mice	Charles River	RRID: IMSR_JAX:000664
Oligonucleotides		
primers for DMS Illumina sequencing	Starr et al.^11^	https://github.com/jbloomlab/SARS-CoV-2-RBD_DMS/blob/master/data/primers/primers.csv
Recombinant DNA		
Expression vectors to produce Spytag-Quartet and Spytag-Alternate Quartet	Hills et al.^12^	N/A
Expression vectors to produce quartet 4a-EABR and quartet 4b-EABR eVLPs	This paper	N/A
Expression vectors to produce SARS-2 RBD-EABR eVLPs	This paper	N/A
mRNA constructs for RBD quartet 4a and RBD quartet 4b	This paper	N/A
mRNA constructs for RBD quartet 4a-EABR and RBD quartet 4b-EABR	This paper	N/A
Expression vectors to produce RBD-Avitag-6xHis	Cohen et al.^7,8^	N/A
Expression vectors to produce Spike-6P-Avitag-6xHis or Spike-2P-Avitag-6xHis	Cohen et al.^13^; Hsieh et al.^78^	N/A
SpyCatcher003-mi3-C-tag	Addgene	Plasmid #159995
Expression vectors of sarbecovirus spikes for making pseudotyped viruses	Cohen et al.^7,8,13^	N/A
Expression vector for BirA	Michael Anaya, Caltech	N/A
Expression vector to produce soluble mouse FcγR2b	Cohen et al.^13^	Uniprot P08101.2 residues 30-208
Expression vector to produce soluble mouse FcγR3	Cohen et al.^13^	Uniprot P08508.1 residues 31-216
Expression vector to produce soluble mouse FcγR4	Cohen et al.^13^	Uniprot A0A0B4J1G0 residues 21-204
Expression vector to produce WA1 RBD-Avitag-6xHis with 1 or more potential N-linked glycosylation sites	This paper	N/A
Software and algorithms		
Graphpad Prism 11.0.0	GraphPad	https://www.graphpad.com/
AntibodyDatabase	West et al.^79^	N/A
Attune NxT Flow Cytometer software	ThermoFisher	www.thermofisher.com
FlowJo 10.5.3	FlowJo	https://www.flowjo.com/
Sony SH800 software v2.1.6	Sony	https://www.sonybiotechnology.com/us/instruments/sh800s-cell-sorter/software/
Adobe Illustrator 2026 30.1	Adobe	https://www.adobe.com/products/illustrator/
Deep mutational scanning processing steps	Greaney et al.^39^	https://github.com/jbloomlab/SARS-CoV-2_Bjorkman_pilot
Swift DMS	Hills et al.^12^	N/A
xPONENT v4.3.309.1	www.diasorin.com	N/A
Python (v3.9.16)	https://www.python.org/	N/A
Pandas (v2.0.3) package	https://pandas.pydata.org/docs/index.html	N/A
NumPy (v1.23.5) package	https://numpy.org/	N/A
Matplotlib (v3.7.1) package	https://matplotlib.org/	N/A
Seaborn (v0.12.2) package	https://seaborn.pydata.org/	N/A
sckit-learn (1.4.2)	https://scikit-learn.org/	N/A
umap-learn (0.5.9.post2)	https://umap-learn.readthedocs.io	N/A
Other		
Nunc MaxiSorp 384-well plates	Sigma	Cat #P6491
mouse IFNg/IL4 double-color ELISpot plates	ImmunoSpot	Cat # mT2003Fp

## Highlights

- Successful conversion of protein-based pan-sarbecovirus vaccine to mRNA formats
- mRNA-encoded mosaic-8 immunogens elicited broad B and improved T cell responses
- SySPEM revealed IgG-subclass-specific epitope signatures across vaccine modalities
- Genetic encoding advances mosaic-8 RBD immunogens toward clinical development
