# Supplementary material for: mRNA delivery of mosaic-8 pan-sarbecovirus RBD vaccines elicits distinct antibody epitope signatures
Source: Cell Rep. Author manuscript; Available in PMC 2026 Jun 22. (PMC13286029; doi:10.1016/j.celrep.2026.117335)
Supplement: 1 [file NIHMS2180997-supplement-1.pdf]

Cell Reports, Volume *45*

Supplemental information

mRNA delivery of mosaic-8 pan-sarbecovirus RBD vaccines elicits distinct antibody epitope signatures

Alexander A. Cohen, Jennifer R. Keeffe, Lusineh Manasyan, Indeever Madireddy, Ange-Célia I. Priso Fils, Kim-Marie A. Dam, Haley E. Stober, Rory A. Hills, Woohyun J. Moon, Paulo J.C. Lin, Mark R. Howarth, Magnus A.G. Hoffmann, and Pamela J. Bjorkman

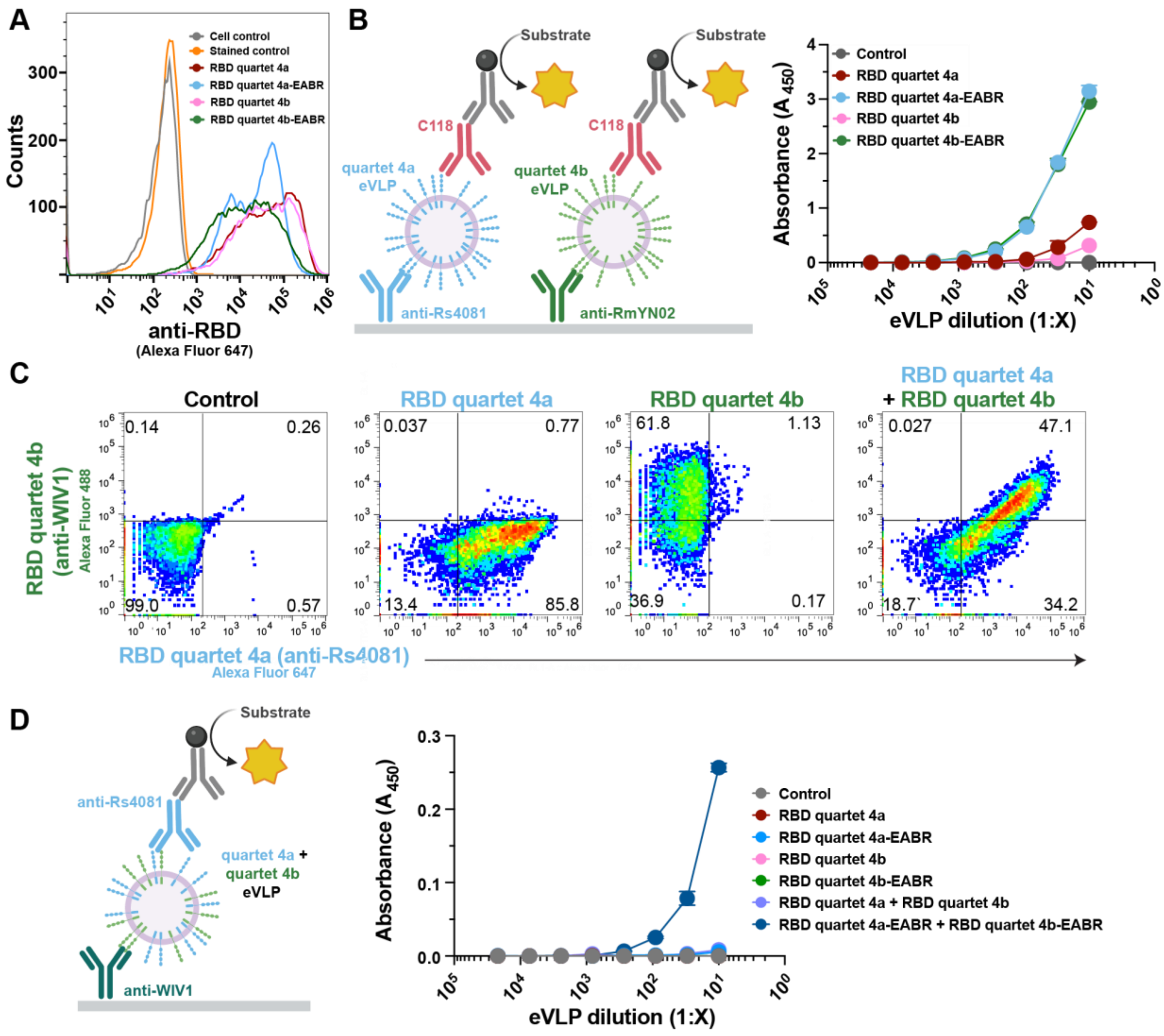

Figure S1. mRNA-encoded dual quartets are presented on cells and eVLPs, Related to STAR Methods.

(A) Flow cytometry using C118, a human pan-sarbecovirus RBD-specific mAb[1,2], demonstrating that transfections of mRNAs encoding EABR and non-EABR versions of RBD quartet 4a or quartet 4b resulted in expression on the surface of transfected cells. Untransfected cells are shown as controls (unstained or stained with C118). (B) Left: ELISA schematic. RBD quartet levels were measured by capturing purified eVLPs with an anti-Rs4081 RBD mAb[3] (recognizes RBD quartet 4a but not quartet 4b) or an anti-RmYN02 RBD mAb[3] (recognizes RBD quartet 4b but not quartet 4a) and detecting with the pan-sarbecovirus RBD-specific C118 mAb.[1,2] Right: ELISA showing that purified single quartet eVLPs purified from supernatants of cells transfected with mRNA encoding RBD quartet 4a-EABR or RBD quartet 4b-EABR displayed RBD quartets on their surface. The mean absorbance of two replicates is shown as a function of eVLP dilution with error bars representing standard deviations. (C) Flow cytometry demonstrating that RBD quartets 4a and 4b were co-expressed on the surfaces of individual cells. RBD quartet 4a was detected using an anti-Rs4081 RBD mAb[3] and RBD quartet 4b was detected using anti-WIV1 RBD mAb.[4] Untransfected cells stained with the anti-Rs4081 and anti-WIV1 RBD mAbs are shown as controls. The percentage of cells in each quadrant is shown. (D) Left: ELISA schematic. Purified eVLPs were evaluated in a sandwich ELISA in which an anti-WIV1 RBD mAb[4] (recognizes RBD quartet 4b but not quartet 4a) was used as a capture Ab and an anti-Rs4081 RBD mAb[3] (recognizes quartet 4a but not quartet 4b) was used for detection. Right: ELISA showing detection of both RBD

quartets 4a and 4b on the surfaces of eVLPs purified from supernatants of cells co-transfected with mRNAs encoding RBD quartet 4a-EABR and 4b-EABR constructs. The mean absorbance of two replicates is shown as a function of eVLP dilution with error bars representing standard deviations. Binding was not detected for purified supernatant samples from untransfected cells (control) or from supernatants from cells transfected with RBD quartet 4a, RBD quartet 4a-EABR, RBD quartet 4b, RBD quartet 4b-EABR, or RBD quartet 4a plus RBD quartet 4b (all data were plotted but some data points near 0.0 A_{450} are obscured by others).

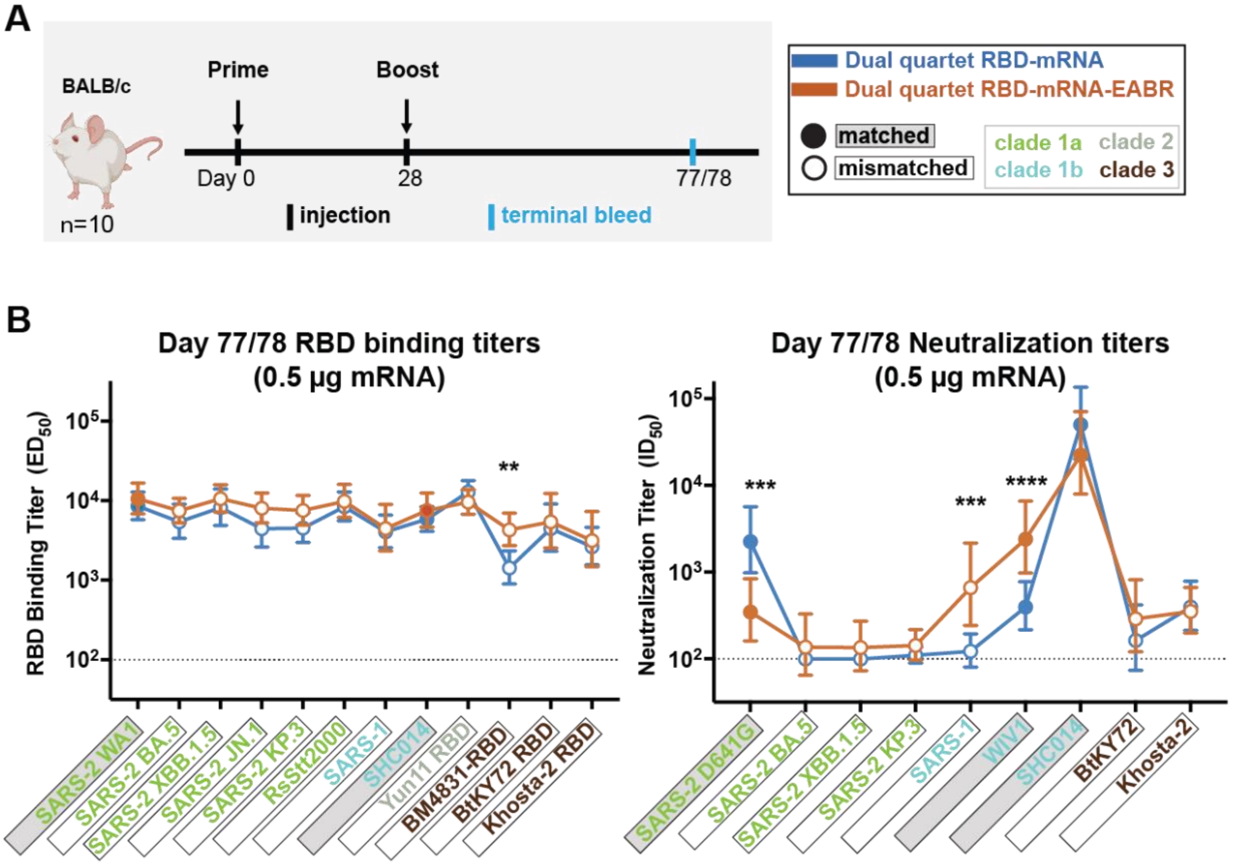

Figure S2. Dual quartet mRNAs elicited cross-reactive Abs, Related to Figure 2.
Data are shown for ELISA and neutralization analyses for terminal bleed serum samples. (A) Immunization regimen. Left: Mice were primed at day 0, boosted at day 28, and samples were collected from a terminal bleed at day 77 or 78. Right: Colors used to identify immunizations and symbols indicating a matched (filled in square data points; gray shading around name) or mismatched (unfilled square data points; black outline around name) sarbecovirus antigen. Sarbecovirus strain names are colored in panel B according to clade. (B) RBD-binding ELISA (left) and pseudovirus neutralization (right) results for serum samples from day 77 or 78 after the prime immunization. Immunogens are shown compared with the cohorts immunized with 0.5 µg of an mRNA-based immunogen. Dashed horizontal lines indicate the limits of detection for each assay. Left: Geomeans of ED_{50} values for animals in each cohort (symbols with geometric standard deviations indicated by error bars) are connected by colored lines. Mean titers against RBDs from indicated sarbecoviruses were compared pairwise across immunization cohorts by Tukey's multiple comparison test with the Geisser-Greenhouse correction (as calculated by GraphPad Prism). Right: Neutralization potencies for serum samples from day 77 or 78 after immunization presented as half-maximal inhibitory dilutions (ID_{50} values) of sera against pseudoviruses from the indicated coronavirus strains. Significant differences between the two cohorts are indicated by asterisks: $p<0.05$ = *, $p<0.01$ = **, $p<0.001$ = ***, $p<0.0001$ = ****.

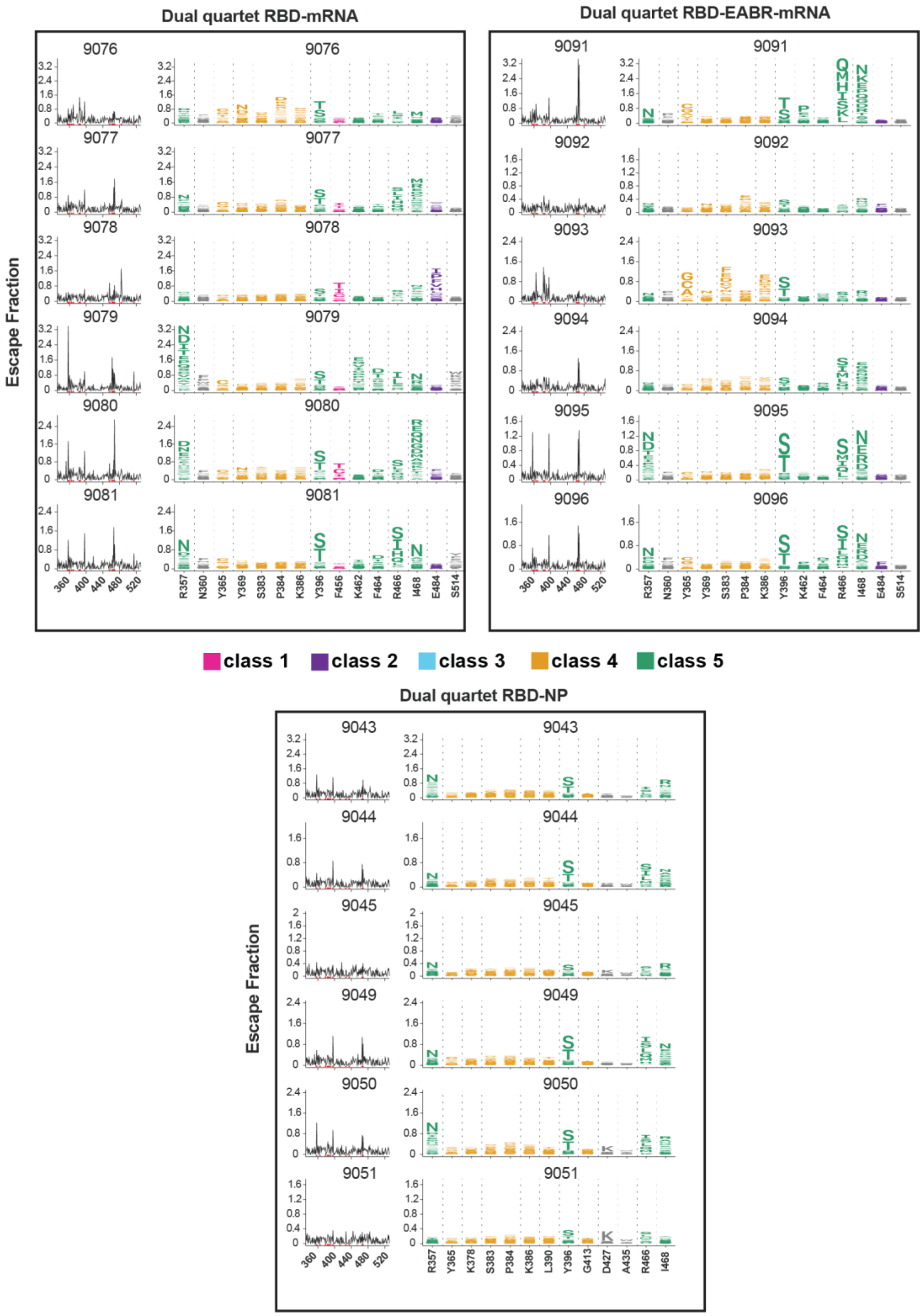

Figure S3. DMS line and logo plots (WA1 RBD library) are shown for individual mice, Related to Figure 4.

DMS line plots (left) and logo plots (right) for results from individual mice (identified by 4-digit numbers) immunized with the indicated immunogens. X-axes of line and logo plots show RBD residue numbers, and y-axes of line plots show the sum of the Ab escape for all substitutions at an RBD residue (larger numbers indicate increased Ab escape). Sites with the strongest Ab escape are shown in logo plots; tall letters represent the most frequent substitutions at a site. Logo plot residues are colored according to RBD epitopes within different classes[2,5-7] as indicated on the legend.

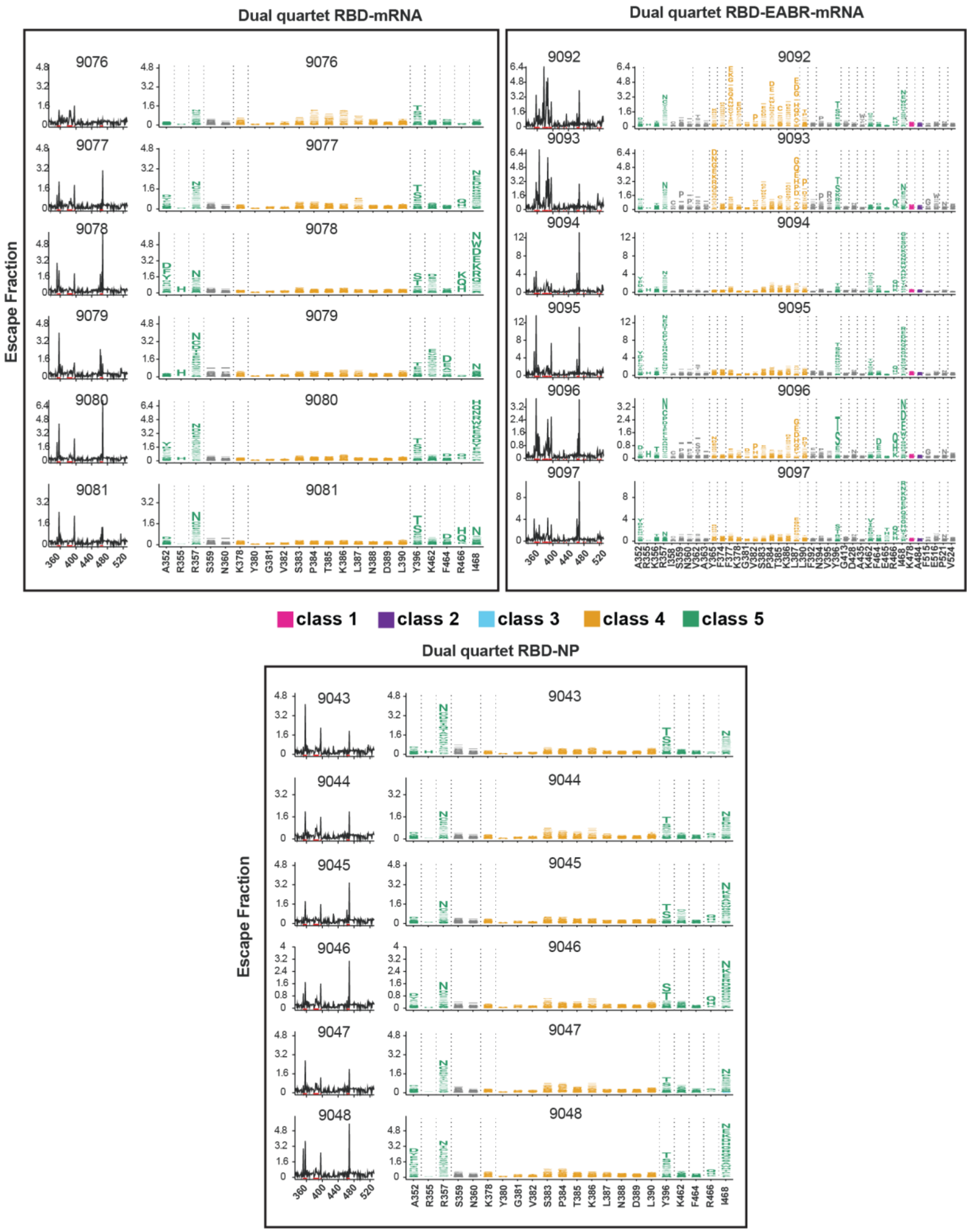

Figure S4. DMS line and logo plots (XBB.1.5 RBD library) are shown for individual mice, Related to Figure 4.

DMS line plots (left) and logo plots (right) for results from individual mice (identified by 4-digit numbers) immunized with the indicated immunogens. X-axes of line and logo plots show RBD residue numbers, and y-axes of line plots show the sum of the Ab escape for all substitutions at an RBD residue (larger numbers indicate increased Ab escape). Sites with the strongest Ab escape are shown in logo plots; tall letters represent the most frequent substitutions at a site. Logo plot residues are colored according to RBD epitopes within different classes[2,5-7] as indicated on the legend.

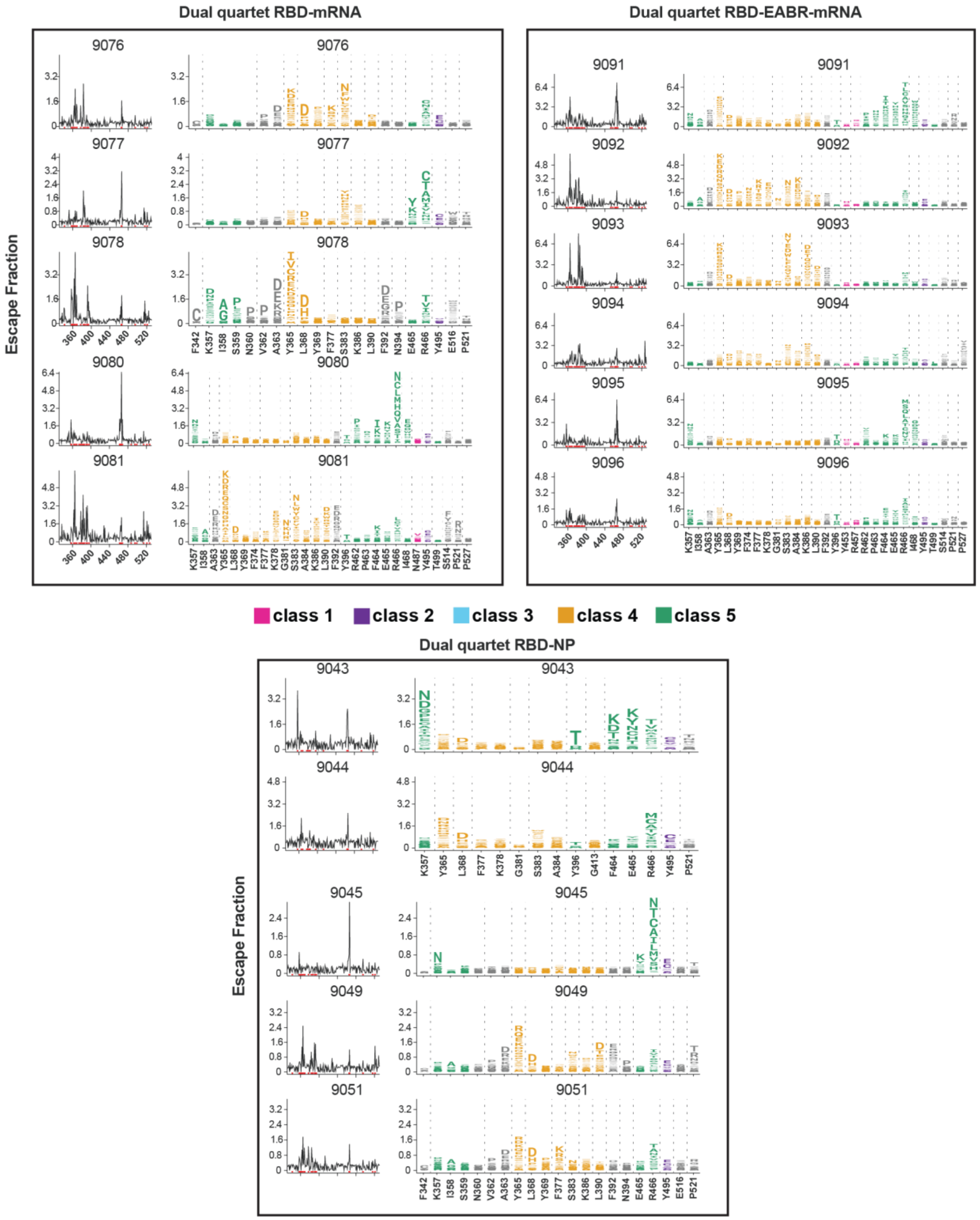

Figure S5. DMS line and logo plots (SARS-1 RBD library) are shown for individual mice, Related to Figure 4.

DMS line plots (left) and logo plots (right) for results from individual mice (identified by 4-digit numbers) immunized with the indicated immunogens. X-axes of line and logo plots show RBD residue numbers, and y-axes of line plots show the sum of the Ab escape for all substitutions at an RBD residue (larger numbers indicate increased Ab escape). Sites with the strongest Ab escape are shown in logo plots; tall letters represent the most frequent substitutions at a site. Logo plot residues are colored according to RBD epitopes within different classes[2,5-7] as indicated on the legend.

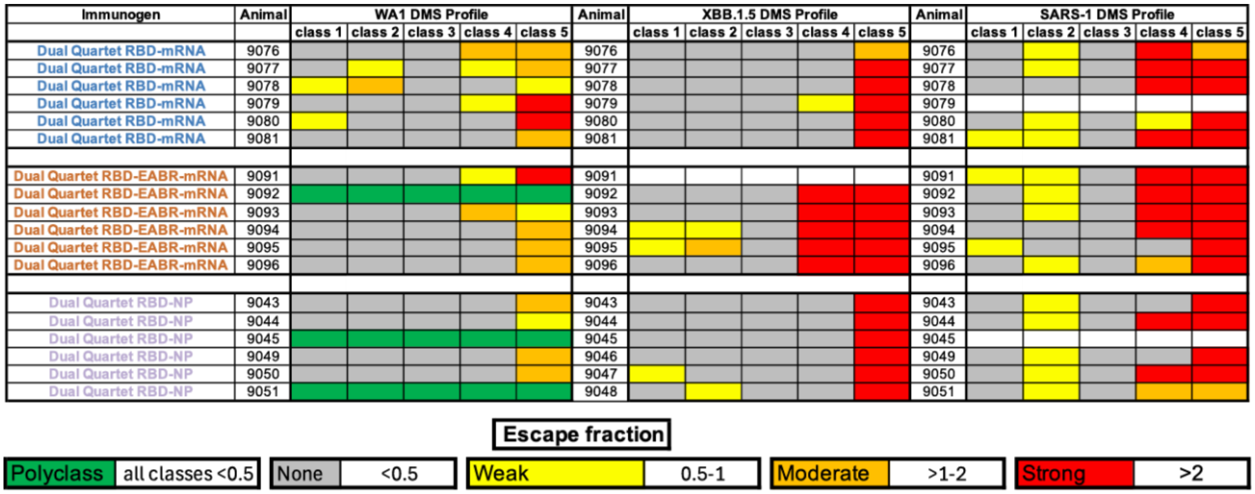

Figure S6. Summary of DMS data showing elicitation of strong Ab responses against class 4 and class 5 RBD epitopes by dual quartet RBD-mRNA immunogens, Related to Figure 4. Results for individual mice are shown with ID numbers indicated. DMS was conducted using a SARS-2 WA1 RBD library (left), a SARS-2 XBB.1.5 RBD library (middle), or a SARS-1 RBD library (right). DMS profiles were classified as polyclass, weak, moderate, or strong as indicated by the maximum escape fraction for any RBD position in each RBD epitope class. Colors and different designations for epitopes were assigned as indicated at the bottom (white entries indicate that no data were available). See also Figure 4 and Figures S3-S5.

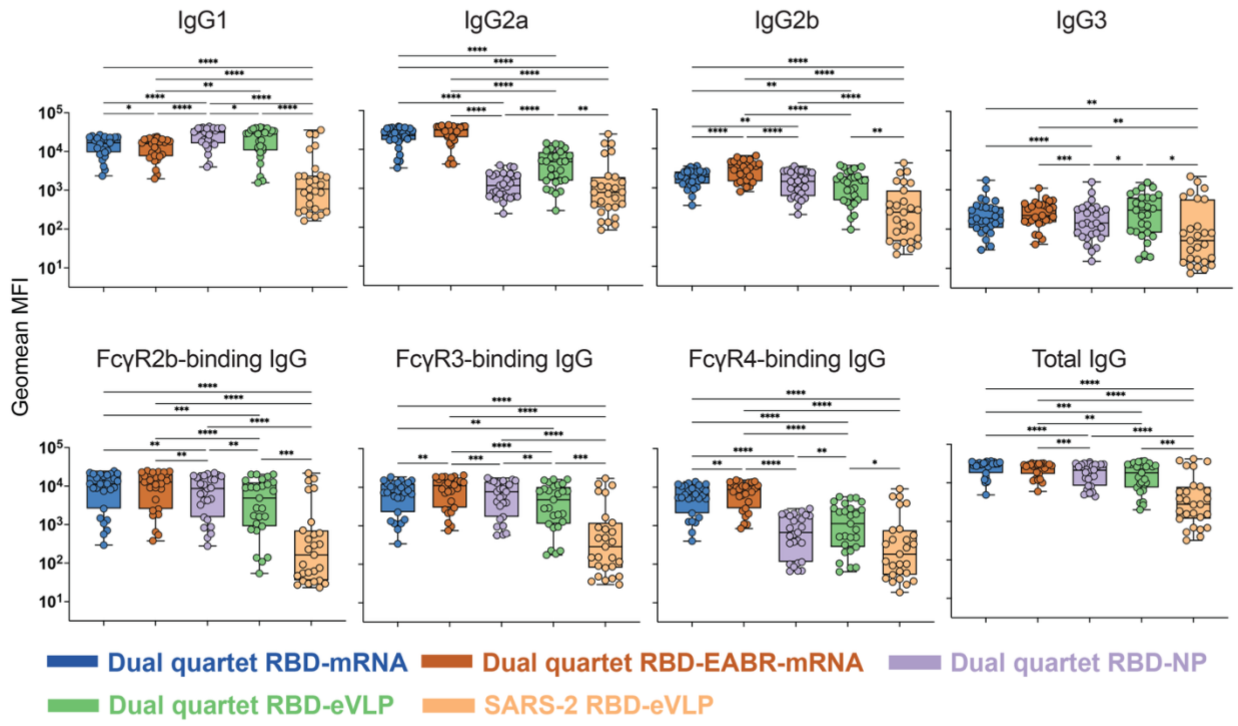

Figure S7. mRNA-encoded dual quartet RBD immunogens elicit balanced IgG subclass and potent FcγR-binding responses, Related to Figure 5.

MFI = Median fluorescent intensity. For IgG1, IgG2a, IgG2b, IgG3, FcγR2b-binding IgGs, FcγR3-binding IgGs, FcγR4-binding IgGs, and total IgG, geomean MFI values of the individual responses shown in panel A for each cohort binding to different spikes or RBDs are represented as points in a box and whisker plot and compared pairwise across immunization cohorts by Tukey's multiple comparison test calculated by GraphPad Prism. Significant differences between cohorts linked by vertical lines in panels B and C are indicated by asterisks: $p<0.05$ = *, $p<0.01$ = **, $p<0.001$ = ***, $p<0.0001$ = ****.

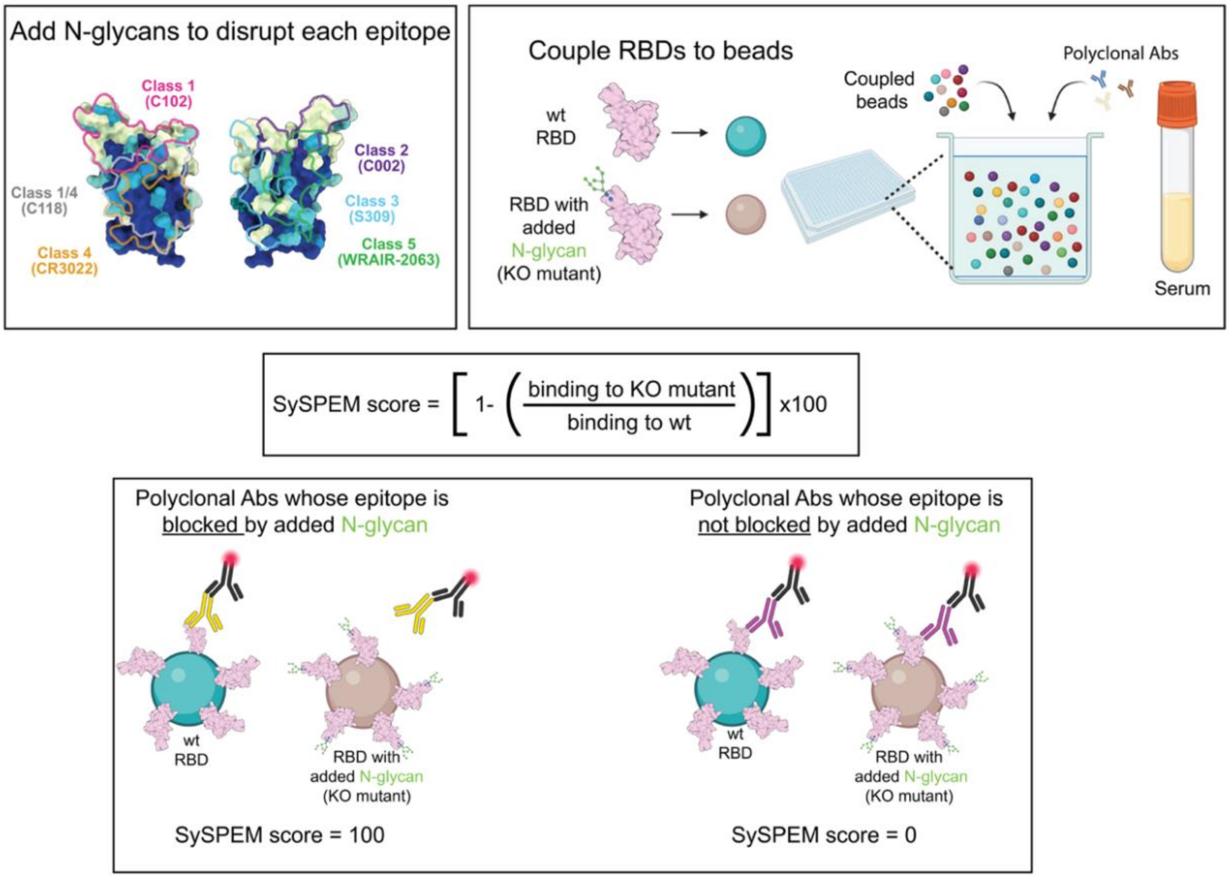

Figure S8. Schematic illustrating the SySPEM approach, Related to Figures 6 and 7.

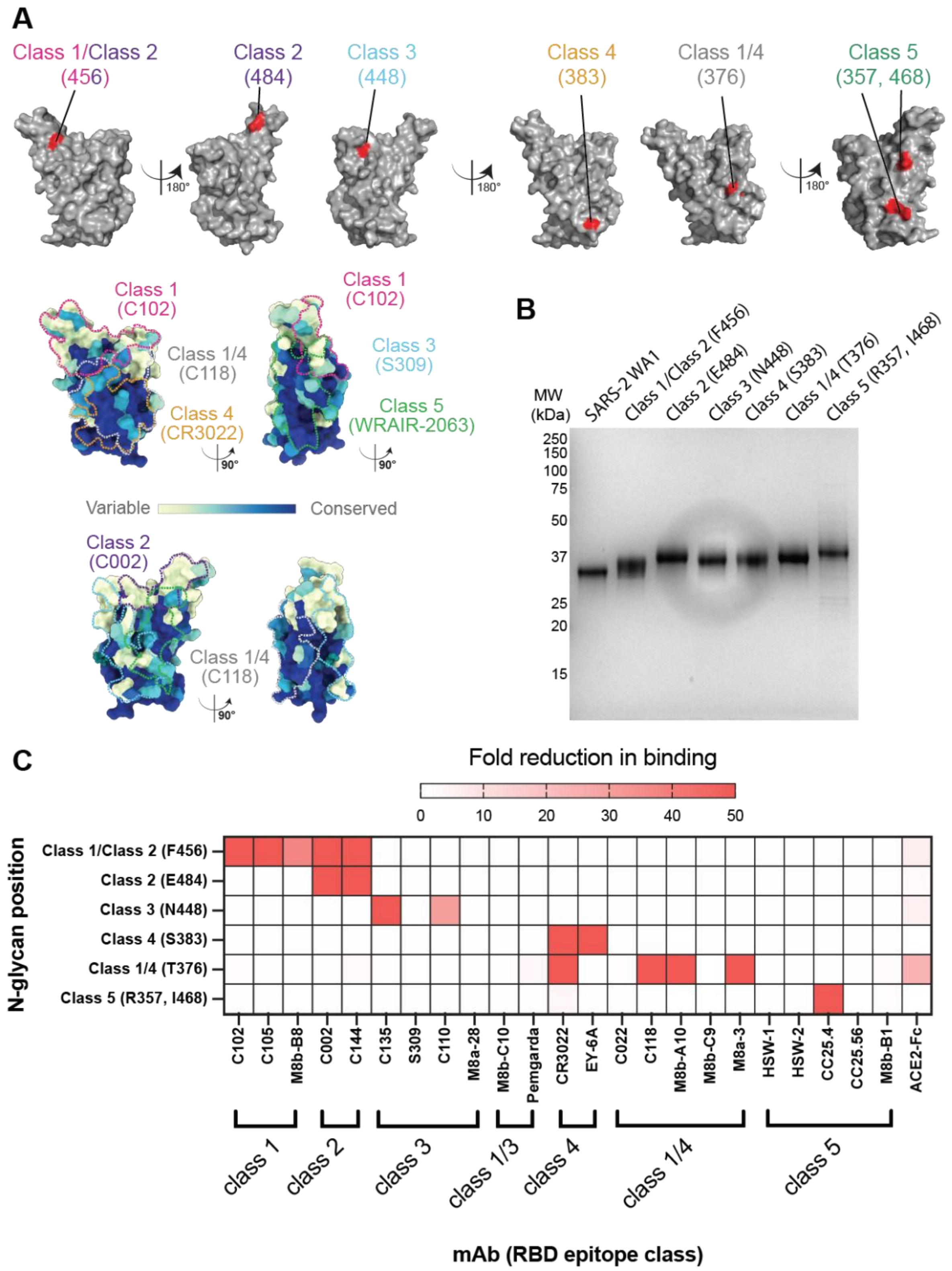

Figure S9. RBD N-glycan mutants used for SySPEM analysis were characterized to ensure glycan addition and proper folding, Related to Figures 6 and 7.

(A) Top: Locations of the added N-glycan(s) in RBD KO mutants, as shown by highlighting of one or more residues that were changed to Asn in the introduced PNGS(s). Bottom: Sequence conservation of 16 sarbecovirus RBDs calculated using ConSurf[8] shown on a surface representation of SARS-2 RBD (PDB 7BZ5). Class 1, 2, 3, 4, 1/4, and 5 anti-RBD Ab epitopes[2,5-7] are outlined in dots in different colors using information from representative structures of Abs bound to SARS-2 spike or RBD (C102: PDB 7K8M; C002: PDB 7K8T, S309: PDB 7JX3;

CR3022: PDB 7LOP; C118: PDB 7RKV; WRAIR-2063: PDB 8EOO). (B) SDS-PAGE analysis of purified wt RBD and RBD KO mutants. Molecular weight marker positions are shown on the left with the molecular weight indicated in kilodaltons. KO mutants are listed with the RBD epitope class affected by the N-glycan addition(s) and residue number(s) of Asn residue(s) to which N-linked glycan(s) were added. (C) Results of ELISA showing ratio of binding of characterized mAbs or human ACE2-Fc[2] to wt RBD versus the RBD KO mutants listed on the left. ELISA EC_{50} values for binding of each reagent to wt RBD and the six RBD KO mutants were derived using , and the fold reduction in binding to each RBD KO mutant was calculated as EC_{50} RBD KO / EC_{50} RBD wt. Classifications of RBD epitopes recognized by the characterized mAbs and ACE-2 Fc are taken from[2-7].

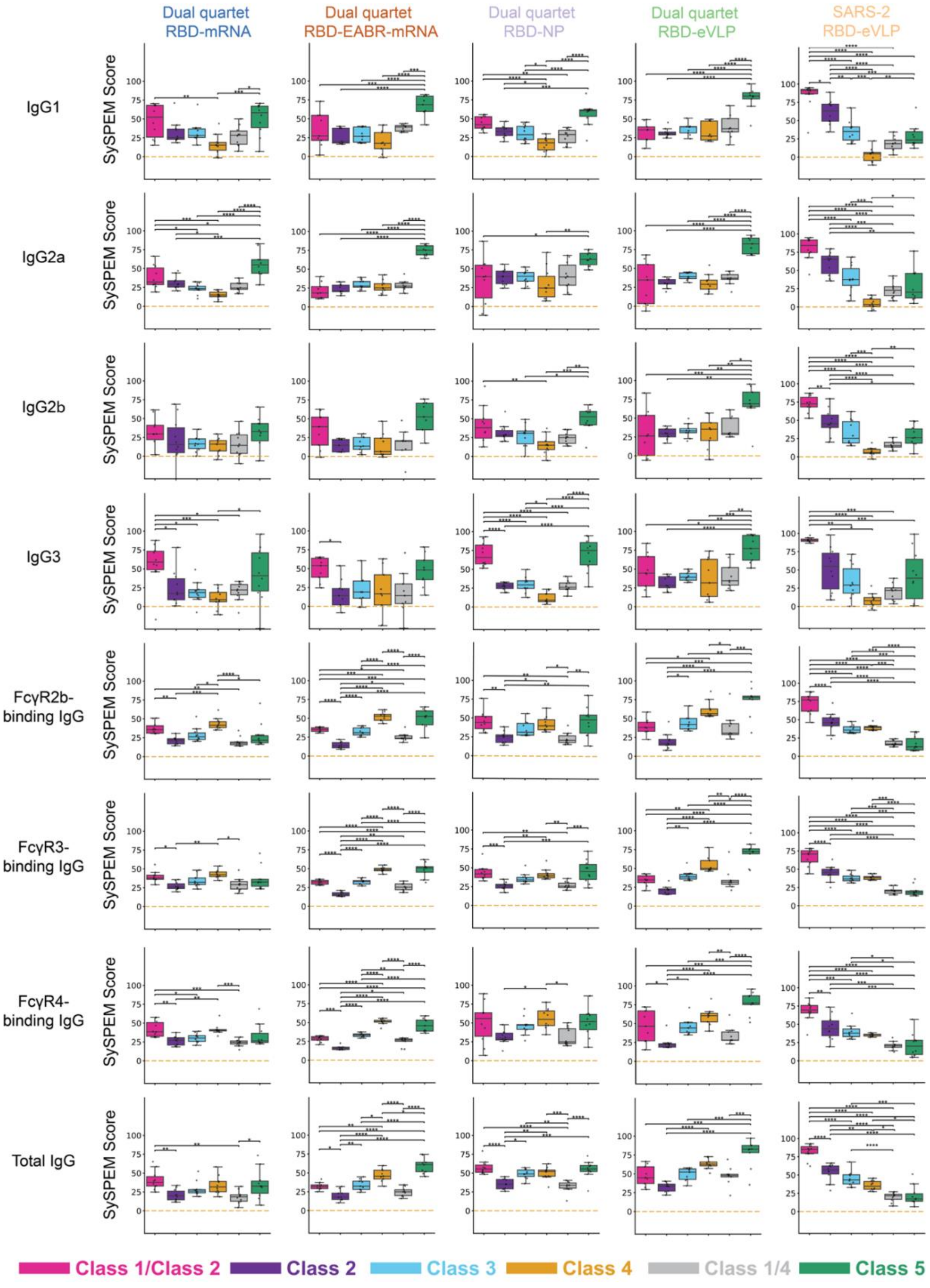

Figure S10. SySPEM score comparisons show distinct epitope profiles across immunogen cohorts, Related to Figures 6 and 7.

SySPEM scores from individual mice in each immunogen cohort (columns) were determined for each IgG class (rows, IgG subclass plus different FcγR-binding IgGs) with statistical comparisons

between targeted epitopes (colors). A SySPEM value of 0 indicates that none of the IgGs in that sample were affected by the glycan addition and therefore the sample did not contain IgGs that recognize that epitope, and a SySPEM value of 100 indicates that all IgGs in that sample recognized that epitope (Figure S7). A SySPEM value between 0 and 100 indicates the proportion of IgGs in a sample that recognized the epitope that was blocked by glycan addition in the RBD KO mutant. Box and whisker plots of SySPEM scores with individual data points representing one mouse are shown. Significant differences between cohorts were calculated using Tukey's HSD posthoc test and linked by vertical lines indicated by asterisks: $p<0.05$ = *, $p<0.01$ = **, $p<0.001$ = ***, $p<0.0001$ = ****. See also Figures 6 and 7.

References

1 Robbiani, D. F., Gaebler, C., Muecksch, F., Lorenzi, J. C. C., Wang, Z., Cho, A., Agudelo, M., Barnes, C. O., Gazumyan, A., Finkin, S., Hagglof, T., Oliveira, T. Y., Viant, C., Hurley, A., Hoffmann, H. H., Millard, K. G., Kost, R. G., Cipolla, M., Gordon, K., Bianchini, F., Chen, S. T., Ramos, V., Patel, R., Dizon, J., Shimeliovich, I., Mendoza, P., Hartweger, H., Nogueira, L., Pack, M., Horowitz, J., Schmidt, F., Weisblum, Y., Michailidis, E., Ashbrook, A. W., Waltari, E., Pak, J. E., Huey-Tubman, K. E., Koranda, N., Hoffman, P. R., West, A. P., Jr., Rice, C. M., Hatziioannou, T., Bjorkman, P. J., Bieniasz, P. D., Caskey, M. & Nussenzweig, M. C. (2020). Convergent antibody responses to SARS-CoV-2 in convalescent individuals. Nature *584*, 437–442.

2 Jette, C. A., Cohen, A. A., Gnanapragasam, P. N. P., Muecksch, F., Lee, Y. E., Huey-Tubman, K. E., Schmidt, F., Hatziioannou, T., Bieniasz, P. D., Nussenzweig, M. C., West, A. P., Keeffe, J. R., Bjorkman, P. J. & Barnes, C. O. (2021). Broad cross-reactivity across sarbecoviruses exhibited by a subset of COVID-19 donor-derived neutralizing antibodies. Cell reports *36*, 109760.

3 Fan, C., Keeffe, J. R., Malecek, K. E., Cohen, A. A., West, A. P., Jr., Baharani, V. A., Rorick, A. V., Gao, H., Gnanapragasam, P. N. P., Rho, S., Alvarez, J., Segovia, L. N., Hatziioannou, T., Bieniasz, P. D. & Bjorkman, P. J. (2025). Cross-reactive sarbecovirus antibodies induced by mosaic RBD nanoparticles. Proc Natl Acad Sci U S A *122*, e2501637122.

4 Fan, C., Cohen, A. A., Park, M., Hung, A. F., Keeffe, J. R., Gnanapragasam, P. N. P., Lee, Y. E., Gao, H., Kakutani, L. M., Wu, Z., Kleanthous, H., Malecek, K. E., Williams, J. C. & Bjorkman, P. J. (2022). Neutralizing monoclonal antibodies elicited by mosaic RBD nanoparticles bind conserved sarbecovirus epitopes. Immunity *55*, 2419–2435 e2410.

5 Barnes, C. O., Jette, C. A., Abernathy, M. E., Dam, K.-M. A., Esswein, S. R., Gristick, H. B., Malyutin, A. G., Sharaf, N. G., Huey-Tubman, K. E., Lee, Y. E., Robbiani, D. F., Nussenzweig, M. C., West, A. P. & Bjorkman, P. J. (2020). SARS-CoV-2 neutralizing antibody structures inform therapeutic strategies. Nature *588*, 682–687.

6 Jensen, J. L., Sankhala, R. S., Dussupt, V., Bai, H., Hajduczki, A., Lal, K. G., Chang, W. C., Martinez, E. J., Peterson, C. E., Golub, E. S., Rees, P. A., Mendez-Rivera, L., Zemil, M., Kavusak, E., Mayer, S. V., Wieczorek, L., Kannan, S., Doranz, B. J., Davidson, E., Yang, E. S., Zhang, Y., Chen, M., Choe, M., Wang, L., Gromowski, G. D., Koup, R. A., Michael, N. L., Polonis, V. R., Rolland, M., Modjarrad, K., Krebs, S. J. & Joyce, M. G. (2023). Targeting the Spike Receptor Binding Domain Class V Cryptic Epitope by an Antibody with Pan-Sarbecovirus Activity. J Virol *97*, e0159622.

7 Cui, L., Li, T., Lan, M., Zhou, M., Xue, W., Zhang, S., Wang, H., Hong, M., Zhang, Y., Yuan, L., Sun, H., Ye, J., Zheng, Q., Guan, Y., Gu, Y., Xia, N. & Li, S. (2024). A cryptic site in class 5 epitope of SARS-CoV-2 RBD maintains highly conservation across natural isolates. iScience *27*, 110208.

8 Landau, M., Mayrose, I., Rosenberg, Y., Glaser, F., Martz, E., Pupko, T. & Ben-Tal, N. (2005). ConSurf 2005: the projection of evolutionary conservation scores of residues on protein structures. Nucleic Acids Res *33*, W299–302.
